# Variable-angle epifluorescence microscopy characterizes protein dynamics in the vicinity of plasma membrane in plant cells

**DOI:** 10.1186/s12870-018-1246-0

**Published:** 2018-03-14

**Authors:** Tong Chen, Dongchao Ji, Shiping Tian

**Affiliations:** 1 Key Laboratory of Plant Resources, Institute of Botany, Chinese Academy of Sciences, Nanxincun 20, Xiangshan, Haidian District, Beijing, 100093 China; 2 University of Chinese Academy of Sciences, Beijing, 100049 China; 3 Key Laboratory of Post-Harvest Handling of Fruits, Ministry of Agriculture, Beijing, China

**Keywords:** Endosomal dynamics, Microdomain, Plasma membrane, Variable-angle epifluorescence microscopy

## Abstract

**Background:**

The assembly of protein complexes and compositional lipid patterning act together to endow cells with the plasticity required to maintain compositional heterogeneity with respect to individual proteins. Hence, the applications for imaging protein localization and dynamics require high accuracy, particularly at high spatio-temporal level.

**Results:**

We provided experimental data for the applications of Variable-Angle Epifluorescence Microscopy (VAEM) in dissecting protein dynamics in plant cells. The VAEM-based co-localization analysis took penetration depth and incident angle into consideration. Besides direct overlap of dual-color fluorescence signals, the co-localization analysis was carried out quantitatively in combination with the methodology for calculating puncta distance and protein proximity index. Besides, simultaneous VAEM tracking of cytoskeletal dynamics provided more insights into coordinated responses of actin filaments and microtubules. Moreover, lateral motility of membrane proteins was analyzed by calculating diffusion coefficients and kymograph analysis, which represented an alternative method for examining protein motility.

**Conclusion:**

The present study presented experimental evidence on illustrating the use of VAEM in tracking and dissecting protein dynamics, dissecting endosomal dynamics, cell structure assembly along with membrane microdomain and protein motility in intact plant cells.

**Electronic supplementary material:**

The online version of this article (10.1186/s12870-018-1246-0) contains supplementary material, which is available to authorized users.

## Background

Like other cellular membranes, plasma membranes (PMs) are highly organized structures subcompartmented in different functional domains, and they encompass a complex set of proteins and lipids essential for cell morphogenesis and patterning [1, 2]. The PM provides an environment in which macromolecules interact efficiently, including the clustering of proteins in oligomeric complexes via protein–protein or protein–lipid interactions, the docking and anchoring of protein complexes for regulatory reactions and other precisely orchestrated processes [1]. Furthermore, the coupling of signal perception with cytoskeletal structures and intracellular second messengers also necessarily involves transduction across the PM. Therefore, the functional dissection of underlying cellular events in the vicinity of the PM, utilizing state-of-the-art techniques, is a priority.

Total internal reflection fluorescence (TIRF), also termed evanescent wave fluorescence, employs evanescent waves to selectively excite fluorophores at the glass / specimen interface and in close proximity to it (100–200 nm) [3, 4]. As a result of total internal reflection (TIR), the evanescent wave forms and propagates immediately beneath the cell surface, exciting only fluorophores within range of the evanescence field. By contrast, fluorescent particles outside of this range remain unexcited, which makes this technique particularly suitable for dissecting molecular and cellular events in close proximity to the PM [4]. TIRF has been used effectively for imaging protein dynamics in animal cells, such as epidermal growth factor dynamics [5], cytoskeletal dynamics [6] and ion channel activity [7]. It depends on the capability of the sample to adhere to the coverslip, however, and to remain within the imaging area defined by the evanescence field.

In plants, however, the plant cell wall imposes a rigid barrier to the real-time tracking of the dynamics of such events with high spatio-temporal accuracy, and this had significantly hampered the efforts of the plant cell research community. Some targets of interest, such as the cells of various plants and fungi, either do not adhere well to coverslips or have thick walls which the evanescence field cannot penetrate well [8, 9], which makes the applications of TIFM in plant and fungal cells more complex. However, the penetration depth of TIRF microscopy depends on the incident angle of illumination, resulting in a range of available depths [3, 4]. Variable-angle epifluorescence microscopy (VAEM) allows laser beam to penetrate the cell wall using a sub-critical angle which was smaller than the critical angle [10]. When the beam is refracted at a steep angle, a slanted “sheet” of light is produced. This technique also produces images with a higher contrast than epifluorescence illumination. Without exception, all single-molecule studies on plant cells using TIRF, VAEM or Highly Inclined and Laminated Optical sheet (HiLo) microscopy [11] are conducted based on this principle.

Here, we present experimental evidence showing the breadth of applications of VAEM in the tracking of protein dynamics involved in organellar dynamics, cytoskeletal structure assembly, membrane microdomain organization and protein motility. These applications span right across the plant science community and, besides revealing the underlying mechanisms of processes linked to the PM, they will undoubtedly stimulate new research directions.

## Methods

### Construction of pABP1:ABP1-YFP binary expression vectors

A genomic fragment of AT4G02980 and the upstreaming sequence of 1500-bp were amplified from genomic DNA samples with primers 5’-CACCAATCTTCATTCTTTACCTGCAC -3′ and 5′- AAGCTCGTCTTTTTGTGATTCTTG-3′, subcloned into pENTR/SD/D-TOPO vectors (Invitrogen), and then sub-cloned into destination vector pMDC107 by LR recombination reaction. *Arabidopsis thaliana* ecotype Columbia wild-type plants were transformed with the fluorescent-tagged ABP1 constructs using the *Agrobacterium tumefaciens*-mediated floral dip method [12]. Kanamycin-resistant transgenic plants were grown on solid medium (1% agar), 1/2 MS medium containing 50 μg/mL kanamycin [13]. 35S:FLOT1a-mCherry is constructed by replacing GFP with mCherry in 35S:FLOT1a-GFP [14].

### Plant materials and growth conditions

Arabidopsis seedlings were maintained as described before [15]. Arabidopsis lines transformed with 35S:FLOT1a-GFP [14], pCLC:CLC-GFP [16], 35S:LTi6a-GFP [17], 35S:GFP-fABD2 and 35S:mCherry-TUA5 [18], 35S:BOR1-GFP [19], pSKU5:SKU5-GFP/*sku5* [20], 35S:StREM-GFP [21], 35S:COBRA-GFP [22], 35S:AtREM1.2-YFP [23], pREM1.2:AtREM1.2-YFP [24] have been described before. Transgenic plants expressing combinations of GFP, mCherry, and YFP fusion proteins were generated by crosspollination, and F1 or F2 generations were used for microscopic observations. Seeds were sterilized with 70% ethanol, 0.1% Triton X-100 / 95% ethanol and plated on half-strength Murashige and Skoog medium (1/2 MS) + 1% agar, stratified for 2–3 days and grown vertically in the chamber with 7000–10,000 lx of illuminance for 16 h per day at 22 ± 2 °C.

### Laser scanning confocal microscopy (LSCM)

Arabidopsis seedlings were imaged with an Olympus FV1000 MPE Multiphoton Laser Scanning Confocal Microscope (60× water-immersion objective; numerical aperture, 1.35). Both GFP and FM4–64 were excited using a 488-nm laser. The fluorescence emission spectra were separated with a 560LP dichroic mirror. GFP fluorescence was collected in the range of 495 to 540 nm, and that of FM4–64 was collected in the range of 570 to 650 nm. mRFP and mCherry were imaged by a 543-nm laser, and the emission fluorescence of mRFP was collected in the range of 580 to 620 nm and that of mCherry in the range of 600 to 650 nm. The colocalization analysis and determination of Pearson’s coefficient were done using the WCIF ImageJ intensity correlation analysis plugin (http://wwwfacilities.uhnresearch.ca/wcif/imagej) [25]. Images acquired were further processed using Adobe Photoshop, version 7.

### Variable-angle epifluorescence microscopy

Four to 5-day-old Arabidopsis seedlings were mounted and observed under an inverted Olympus IX71 microscope equipped with the Andor TIRF illuminator. For sample preparation, seedlings were immersed in 1/2 MS on a slide (BRAND Gmbh, Wertheim, Germany; *n*, 1.52 ± 0.01; thickness, 0.13–0.17 mm). Another cover glass was placed on top of the sample, and this sandwich was pressed gently to tightly attach the seedling to the glass surface. The Andor attachment, which lies upstream of the objective, consists of a set of adjustable reflective mirrors controlling the path of the laser into the 100 × oil-immersion TIRF objective (Olympus; numerical aperture = 1.45). The 473−/561-nm laser line from a diode laser (Changchun New Industries Optoelectronics Technology) was employed to excite GFP and mCherry fluorophores respectively. Fluorescence signals were collected by the objective and passed through two filters, a BA 510IF long-pass filter (Chroma) and a HQ525/50 band-pass filter (Chroma), before being directed using a back-illuminated EMCCD camera (ANDOR iXon DV8897D-CS0-VP, Andor Technology) and high-quality filters (band-pass 525/545 and 609/654 nm).We set the EM-gain of EMCCD camera at 433 throughout all imaging experiments unless otherwise indicated. Images were acquired with 100-ms exposure time (unless otherwise indicated) and analyzed with Image J software (NIH). The incident angle was adjusted and calibrated according to the methodology reported by Wan et al. [10], during the experiments, and the details were also included in Additional file 1. The co-localization analysis for dual-color observations was performed by using WCIF ImageJ intensity correlation analysis plugin (http://www.facilities.uhnresearch.ca/wcif/imagej/colour_analysis.htm) [25].

Skewness analysis was used to obtain the extent of bundling, and the filament density was calculate as the percentage of occupancy of GFP-fABD2 signal in each micrograph [26, 27]. Micrographs were analyzed with ImageJ (http://rsb.info.nih.gov/ij/) using Higaki’s macro available at [26, 27].

### Pharmacological studies with inhibitors

TyrA23 and isobaxen were purchased from Sigma-Aldrich. The inhibitors were dissolved in 100% DMSO as stock solutions and further diluted in 1/2 MS for VAEM imaging on epidermal cells. The final concentration of DMSO was 0.1% or even lower in all working solutions. Four- to 5-d-old vertically grown seedlings were transferred from 1% agar plates to a well of a 12-well plate containing 4 mL of final working concentration in 1/2 MS. After the indicated incubation duration, seedlings were transferred to a glass slide with 100 μL inhibitor solution, covered with a glass coverslip for imaging as above.

### Single particle tracking and data analysis

Single-particle tracking was accomplished using spatially and temporally global particle assignment, with MATLAB as detailed in [28, 29], and only tracks with lengths of more than ten frames were kept for further analysis. For each trajectory, the mean square displacement (MSD) was computed from the formula:

$$ \mathrm{MSD}\left(\mathrm{t}\right)=\frac{1}{\mathrm{L}-\mathrm{n}}{\sum}_{\mathrm{s}=0}^{\mathrm{L}-\mathrm{n}-1}{\left(\mathrm{r}\left(\mathrm{s}+\mathrm{n}\right)-\mathrm{r}\left(\mathrm{s}\right)\right)}^2 $$

Where *n* = *t*/*△t*, L is the length of the trajectory (number of frames) and *r*(*s*) is the two-dimensional position of the particle in the frame *s* (*s* = 0 corresponds to the start of the trajectory) [30]. To determine the diffusion coefficient from a trajectory, a line was fit to the MSD with *n* running from 1 to the largest integer less than or equal to *L*/4 [31]. The diffusion coefficient of the particles was calculated by linear fitting (MSD = 4Dt + c) to MSD versus time (MSD-t), and the distribution of the diffusion coefficients was plotted in a histogram with logarithmically spaced bins. The acquired data were further subjected to Gaussian fitting, the position of the peak being considered the characteristic diffusion coefficient of each population. Quantification of the colocalization of CLC and AtFLOT1a was performed according to the protein proximity index method [29, 32, 33].

### Fluorescence correlation spectroscopy (FCS)

FCS analysis was carried out on a Leica TCS SP5 FCS microscope equipped with a 488-nm argon laser, in-house coupled correlator and Avalanche photodiode, using the point-scanning mode. The laser focus was placed at the plasma membrane of the cells, thus the fluctuations in fluorescence intensity was recorded during the diffusion of SKU-GFP molecules in and out the focal volume. Afterwards, the SKU5-GFP density was calculated according to the procedures described previously [4].

## Results

### VAEM is ideal for dissecting organellar identities and dynamics

**Fig. 1:**
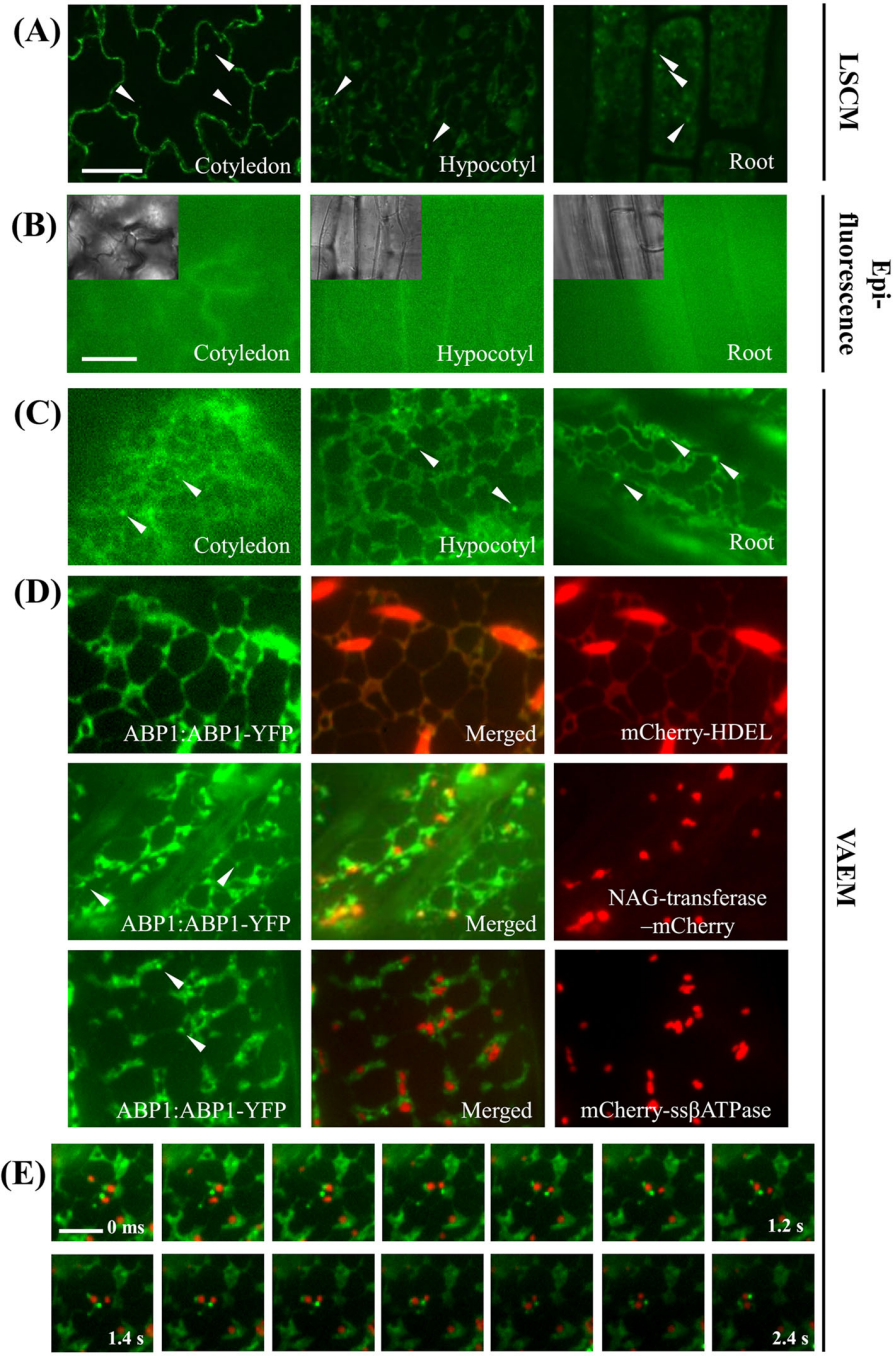
Dual-color VAEM fluorescence co-localization with organelle markers is ideal for dissecting protein localization and dynamics in the cell cortex, taking Auxin Binding Protein 1 (ABP1) as an example. The punctate structures in VAEM images were tracked by using spatially and temporally global particle assignment with MATLAB. The incident angles were between 63.03° and 66.64° for HDEL-GFP seedlings, corresponding to penetration depths at 120 nm (66.64°) – 250-300 nm (63.03°) respectively, depending on the practical conditions for the adherence of seedling. **a** Transgenic Arabidopsis line expressing ABP1:ABP1-YFP (ABP1:ABP1-YFP / Col-0) showing subcellular localization to plasma membrane and intracellular structures, circles indicates punctate structures tagged by YFP fluorescence; **b** Transgenic Arabidopsis line expressing ABP1:ABP1-YFP showing blurred fluorescence, corresponding bright field images were shown in the inlets; **c** Transgenic Arabidopsis line expressing ABP1:ABP1-YFP showing uniformed distribution to ER and punctate structures under VAEM; **d** The punctate structures partly co-localize to Golgi apparatuses but do not co-localize to mitochondria; **e** Some ABP1-YFP tagged punctate structures frequently co-diffuse with Golgi apparatuses. The time series are shown for every 4 images. Bars = 200 μm (**a**), 7 μm (**b**-**d**), 5 μm (**e**). Interval between frames = 200 ms

Since the PM and intracellular compartments comprise a functionally interrelated network, we were keen to investigate the robustness for VAEM in tracking the dynamics of endomembrane components. In this regard, we studied auxin binding protein 1 (ABP1). Initially identified by its capacity to bind auxin and affect PM hyperpolarization, the functions of ABP1 are still under debate [34]. To obtain experimental cues for investigating the subcellular localization of ABP1, we generated an ABP1-YFP transgenic line under the control of its native promoter and examined the localization of ABP1 in the cotyledons under LSCM and VAEM, respectively. In accordance with previous reports, as visualized under LSCM, ABP1 was mainly localized to the PM and to highly dynamic intracellular structures (Fig. 1a). Unfortunately, it was not easy to discern under LSCM the intracellular structures that were labeled with fluorescence, but under VAEM it was reasonably clear that they were associated with the endoplasmic reticulum (ER) and some mobile intracellular compartments. As the expression level under the native promoter was low, the images obtained with wide-field epifluorescence could not clearly differentiate between areas with or without fluorescence (Fig. 1b). In contrast, we were gratified to discover that the intracellular compartments defined by ABP1-YFP fluorescence were well correlated with the mCherry-HDEL tagged ER in the time-series, they were partly co-localized with Golgi apparatuses (N-acetyl glucosaminyl (NAG) transferase–mCherry), but not with mitochondria markers (mCherry-ssβATPase) (Fig. 1c-d). Notably, some bright punctate structures, possibly vesicles or other endosomal structures, could be observed adjacent to the ER or transported along the extended ER tubules (Fig. 1d), further implying substantial roles for these ABP1-labelled structures in ER-endosome association. It was also found that the punctate structures co-diffused with Golgi apparatuses in the time series (Fig. 1e), further suggesting the involvement of ABP1 in endosomal trafficking and endocytic/exocytic activities. As shown in Fig. 1, under VAEM the low background fluorescence allowed us to follow easily at high temporal resolution (200 ms) the ER dynamics in cotyledon epidermal cells as characterized using fluorescence proteins, which was not possible using conventional LSCM. Similarly, other functionally correlated organelles displaying high mobility, namely Golgi apparatuses and mitochondria, were also visualized with NAG transferase-GFP and mCherry-ssβATPase transgenic lines (Additional file 2: Figure S1, Additional files 3 and 4) [35, 36].

In addition to the imaging in cotyledon epidermal cells, VAEM is also applicable to epidermal cells of other tissues. We further used the transgenic HDEL-GFP line to follow ER dynamics in hypocotyl epidermal cells, root epidermal cells, young rosette leaves and stomata respectively, further demonstrating the wide applicability of VAEM in plant cell researches. Similarly, hypocotyl epidermal cells were readily imaged due to their relatively big size and planar shape (Additional file 2: Figure S2). It can be found the ER tubules were distributed in a lower density in root. However, as part of the tubules were not in the imaging plane, the variable and non-planar shape of leaf pavement cells only allow us to observe ER dynamics in limited areas closely adhering to the slide in these cells (Additional file 2: Figure S1 and Additional file 5). As cell wall and specimens of larger sizes may inevitably pose a physical barrier in observations on plant cells, freshly detached tissues are always preferred to obtain better efficacy in adhering the specimen closely to the slide, which is particularly applicable to observations in cotyledon and young leaves.

### Endocytic events and intracellular trafficking can be followed under VAEM

**Fig. 2:**
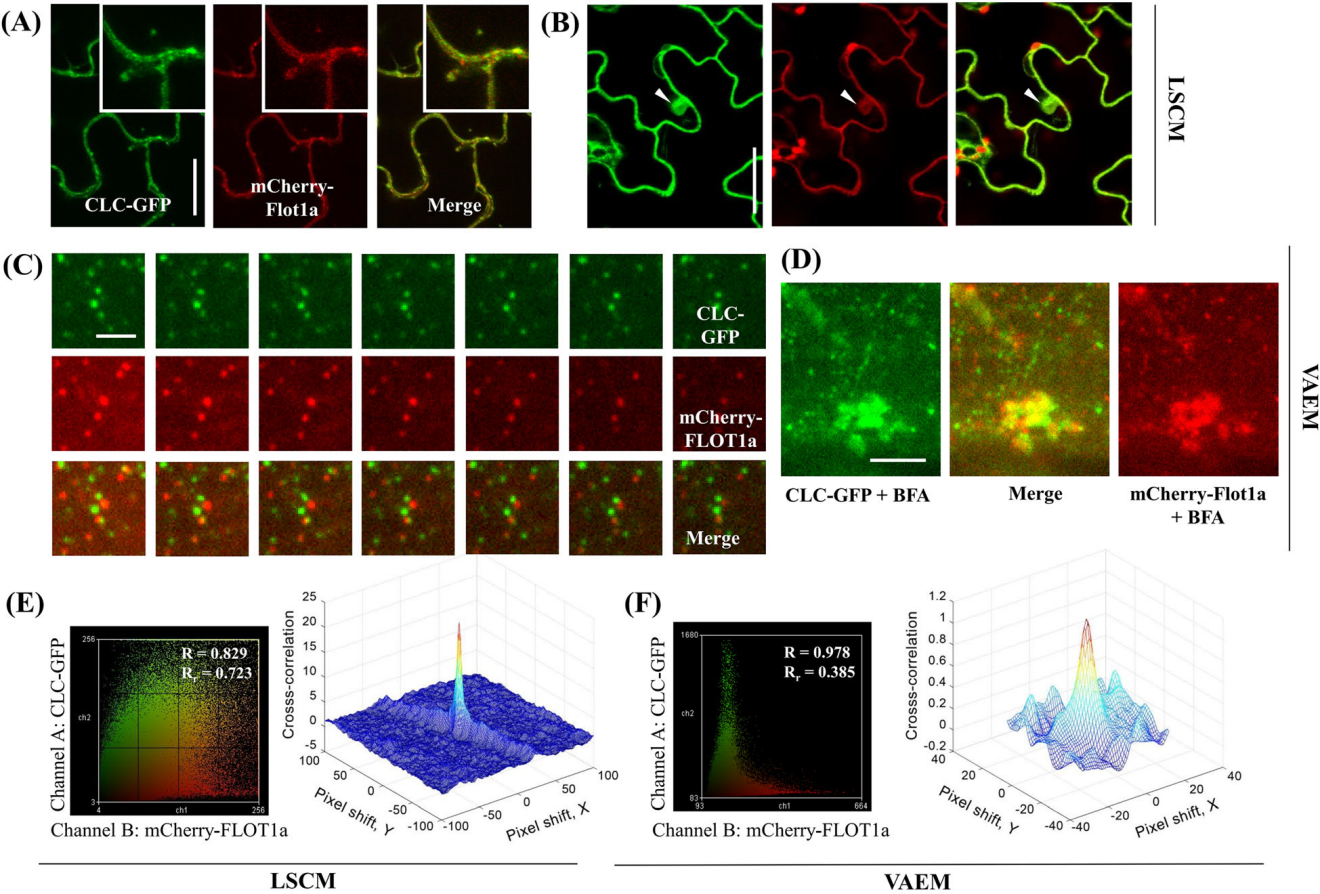
VAEM allows for reliable co-localization analysis on protein candidates at single particle level. **a** Clathrin light chain (CLC) -GFP and mCherry-FLOT1a both localized to PM and intracellular punctate structures under LSCM; The close-up inlets showed that CLC-GFP and mCherry-FLOT1a colocalized at PM and intracellular structures; **b** BFA treatment induced both CLC-GFP and mCherry-FLOT1a to accumulate into BFA compartments, indicating both clathrin and FLOT1a were involved into active endocytic events; **c **Transgenic Arabidopsis line co-expressing CLC-GFP and mCherry-FLOT1a showing characteristic localization to largely separated punctate structures; **d** VAEM observations on BFA-induced compartment formation. **e** Co-localization analysis using ImageJ intensity correlation analysis plugin and Protein Proximity Index (PPI) analysis on the LSCM images (**a**) indicated clathrin and FLOT1a were co-localized in a high percentage; **f** Co-localization analysis using ImageJ intensity correlation analysis plugin and PPI analysis on the VAEM images (**c**) indicated clathrin and FLOT1a were associated rather than co-localized. Bars = 20 μm (**a**-**b**), 3 μm (**c**-**d**); Interval between frames = 200 ms

Given that, as the smallest units, endocytic vesicles play pivotal roles in connecting different endosomal components, the role of clathrin in internalizing extracellular substances has been well investigated, as it is assembled at the PM and initiates endocytic events [37]. In the present study, the TIRF system was equipped with a micrometer allowing continuous lateral adjustment of the spatial filter assembly, such that the position of the beam at the back aperture of the objective could be modulated to switch between TIRF and epifluorescence modes. Under epifluorescence illumination, only blurred fluorescence was found to be associated with the PM, whereas, under VAEM, clathrin light chain (CLC)-GFP formed discrete foci at the PM or were organized into small clusters, contributing to the formation of an endocytic complex at the site (Fig. 2a and Additional file 2: Figure S3). The CLC-GFP foci at the PM could be easily distinguished, though CLC-GFP may also label some brighter, out-of-focus intracellular structures, corresponding to different incident angles and penetration depths [ranging from approximately 100 nm (67.73°) and 130 nm (65.69°)] (Additional file 2: Figure S3 and Additional file 1, indicated with arrowhead), possibly Golgi apparatuses or trans-Golgi network (TGN). When the incident angle of the laser was decreased from subcritical angles towards zero degrees, the foci were lost in the background signal arising from organelle-localized CLC-GFP and cytoplasmic CLC-GFP signals. Similarly, AtFLOTTILIN1a (FLOT1a) is identified as another candidate involved in endocytic activities independent of the clathrin-mediated pathway [14]. As it has been reported previously to be a potential raft-located protein [14], the dynamics of FLOT1a were also examined under VAEM. As expected, mCherry-FLOT1a was found to be prominently associated with the PM and was organized into punctate structures. The FLOT1a-positive punctate structures were dynamic in the vicinity of the PM, but in contrast to those observed in the CLC-GFP lines, some of the puncta were laterally mobile (Fig. 2c and Additional file 6), possibly as individual structures budding off from the PM and entering the cytoplasm. In the presence of BFA, a specific pharmacological agent capable of inhibiting COPI vesicle formation at the Golgi apparatus [38], CLC-GFP and mCherry-FLOT1a were largely colocalized to the BFA-induced compartments during their formation, whereas there were still some apparently associated green and red puncta in the cytosol (Fig. 2d).

**Fig. 3:**
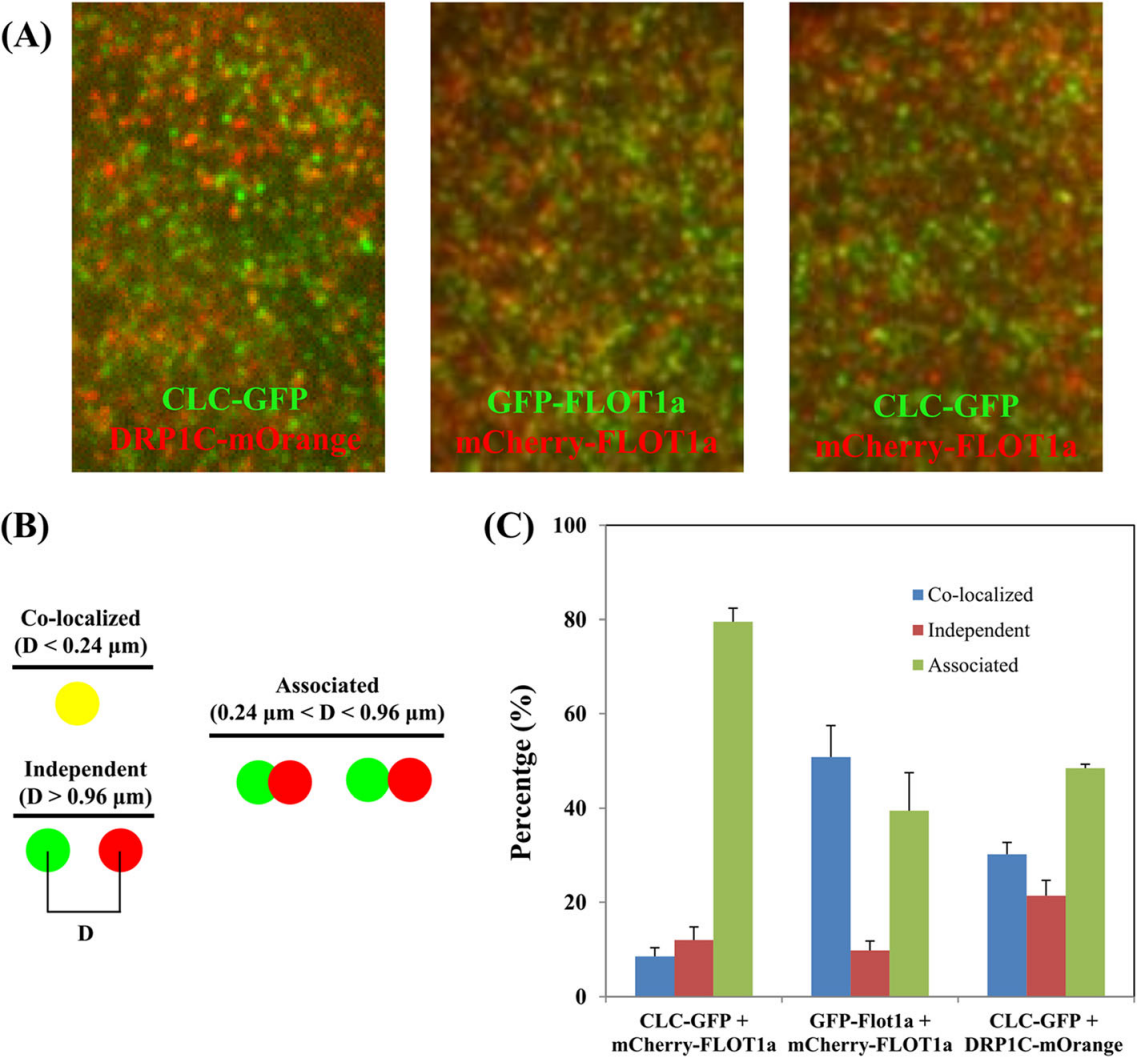
Co-localization analysis on proteins of interest using VAEM-based single particle tracking. **a** Transgenic Arabidopsis line co-expressing CLC-GFP / DRP1C-mOrange, GFP-FLOT1a / mCherry-FLOT1a and CLC-GFP / mCherry-FLOT1a were subjected to VAEM followed by single particle tracking and calculation on the distance between the captured punctate structures labelled by dual colors. **b** We classified the resulting distances into three categories: (i) colocalized: a distance between two centers that was below the resolution limit of the objective lens (0.24 μm in this study); (ii) associated: a distance less than the sum of the radii of two punctate structures (< 0.96 μm in this study); and (iii) independent: a distance larger than the sum of the radii of two punctate structures (> 0.96 μm). D stood for the distance between the centers of two adjacent puncta for analysis. **c** Percentages of co-localized, associated and independent puncta for the protein candidates

As both CLC-GFP and mCherry-FLOT1a are sensitive to brefeldin A (BFA) treatments (Fig. 2a-b), we were curious to examine whether CLC-coated vesicles and FLOT1a-tagged vesicles were colocalized following internalization. Therefore, fluorescence-based co-localization analysis was employed to study the functional correlation of both proteins in terms of their specific cellular location. We co-expressed CLC-GFP and mCherry-FLOT1a and then analyzed their spatial correlation using LSCM images and VAEM time-series images, respectively. Both FLOT1a and CLC have been reported previously as membrane associated proteins and, as shown in Fig. 2a, both CLC-GFP and mCherry-FLOT1a were found to be localized to the PM and to some intracellular structures. Further close-up LSCM observations indicated that CLC-GFP and mCherry-FLOT1a puncta were co-localized or closely associated with each other, correlating well with the high overlap coefficient, R_r_ = 0.723, as shown in the scatter plots (Fig. 2a and e). In contrast to the LSCM findings, however, the VAEM time series showed that in fact most of the CLC-GFP and mCherry-FLOT1a puncta were not co-localized (Fig. 2c). As a result, the overlap coefficient R_r_ was 0.385 from the calculation using the time-series images (Fig. 2f, ten sequential frames in the present study), indicating a low percentage of co-localization of these two protein candidates and further suggesting that VAEM time-series can provide a more reliable co-localization analysis of intracellular structures. As a quantitative measure of colocalization emphasizing that colocalization occurs at the length scale of the resolution of the microscope [33], Protein Proximity Index (PPI) was also calculated for comparison, as shown in the 3D cross-correlation plots (Fig. 2e and f), [PPI = 0.25 ± 0.05 (GFP channel) and 0.41 ± 0.06 (mCherry channel) for VAEM, where PPI = 0.53 ± 0.17 (GFP channel) and 0.72 ± 0.22 (mCherry channel) for LSCM]. Moreover, transgenic *Arabidopsis* lines co-expressing CLC-GFP / mCherry-FLOT1a, GFP-FLOT1a / mCherry-FLOT1a and CLC-GFP / dynamin related protein 1C (DRP1C)-mOrange generated by crossing, were each subjected to VAEM observations followed by single-particle tracking (SPT) and calculations were made of the distances between captured dual-color labeled punctate structures (Fig. 3a). In the control (GFP-FLOT1a and mCherry-FLOT1a), about 50% of the GFP signal was classified as co-localized and more than 40% was classified as associated. Among the protein candidates examined, CLC were found to be either co-localized (about 30%) or associated (50%) closely with DRP1C, which was consistent with their synergistic actions in vesicle formation and internalization; furthermore, about 80% of the CLC-positive puncta were classified as associated with mCherry-FLOT1a, whereas only 8% or 12% of the GFP signals, respectively, was co-localized to or independent to mCherry-FLOT1a puncta, further confirming the applicability of VAEM-based SPT for the co-localization analysis of putatively correlated proteins (Fig. 3b-c).

### VAEM observations on coordinate behaviors in cytoskeletal dynamics

**Fig. 4:**
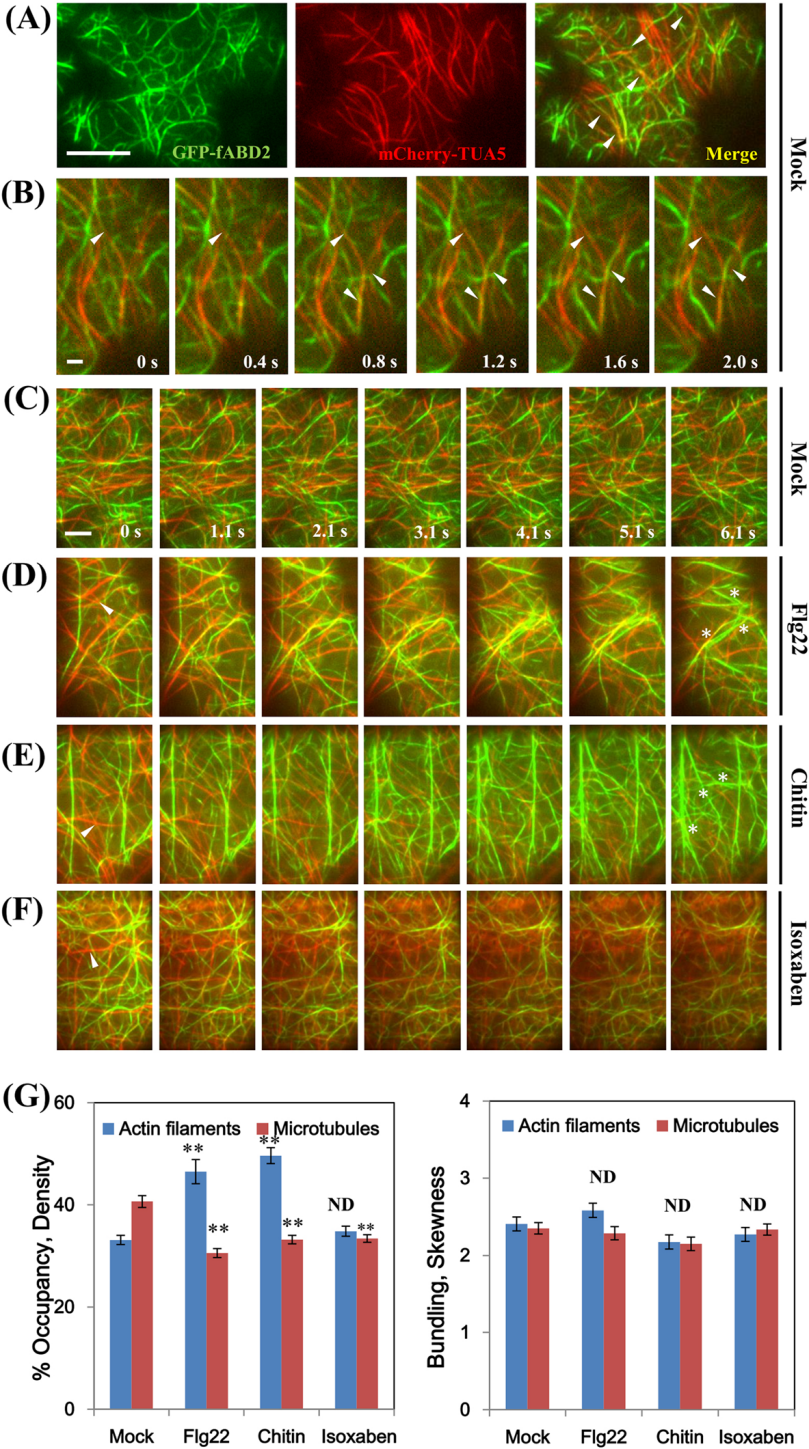
PAMP significantly altered cytoskeletal architecture and dynamics as revealed by dual-color VAEM. To investigate whether the two cytoskeletal structures interact coordinately, seedlings expressing green fluorescent protein (GFP)-F-actin binding domain of fimbrin2 (fABD2) and mCherry-α-tubulin 5 isoform (TUA5) were generated by crossing. LSCM and VAEM were carried out on the seedlings respectively. **a** AFs and MTs in mock-treated epidermal cells from the wild-type cells, arrows indicate actin fragments reside in transient coincidence with MTs; **b** Selected frames from a time series of a GFP-fABD2 and mCherry-TUA5 dual-labeled line; **c** AFs and MTs in the wild-type epidermal cells. **d** to **e** AFs appeared to be more abundant after flg22 and chitin treatment, while the transverse MT organization gradually changed to longitudinal fragments. Epidermal cells treated with 1 μM flg22 or 10 μM chitin had a significant increase in AF abundance compared with mock-treated seedlings. **f** To address the specificity for the elicitor-triggered reorganization of cytoskeletal components as above, 50 μM isoxaben, a cellulose synthesis inhibitor, was applied to treat the seedlings. The MTs were dramatically disordered following incubation while AFs did not show significant changes in density and intensity. **g** Average filament density (percentage of occupancy) analysis was performed on images collected from epidermal cells in the cotyledons. When compared with mock-treated cells, the AF density was significantly increased after 1 μM flg22 and 10 μM chitin treatments; in contrast, MT density decreased significantly after chitin and flg22 treatment, while isoxaben treatment significantly decrease MT density without affecting AF density; The extent of filament bundling, or skewness, was measured. No significant differences were observed among treatments. Values given were mean ± SE. (*n* = 100 cells from 10 cotyledons per treatment; ** *P* < 0.001; ND, no significant difference; Student’s t-test). Bars = 10 μm. Interval between frames = 200 ms, “0 s” referred to the time point at 5 min after corresponding treatment

Luckily, VAEM imaging using subcritical angles also allowed the tracking of cortical actin filaments (AFs) labelled with the GFP-tagged F-Actin Binding Domain of Fimbrin2 (GFP-fABD2) (Fig. 4, Additional file 2: Figure S4, and Additional file 7). As shown in Additional file 2: Figure S4, extensive arrays of longitudinal actin bundles and a dynamic network of AFs could be observed in elongated epidermal cells of the hypocotyls. Several filaments rapidly became bundled into single filaments, but also individual filaments participated in the creation of multiple bundles, all utilizing the same zipper-like motion (indicated by arrows). To follow the dynamics of microtubules (MTs) in epidermal cells, epidermal hypocotyl cells of plants expressing mCherry-α-tubulin 5 isoform (TUA5) were also imaged. The MTs were clearly visible at subcritical angles, showing all the typical configurations previously described for wild-type seedlings: basket, longitudinal, oblique and transverse array (Additional file 2: Figure S5). In time-lapse images of mCherry-TUA5-expressing cells (Additional file 8), though microtubule growth was apparent, microtubules did not show obvious assembly and disassembly events at a temporal resolution of 200 ms.

Simultaneous tracking of both types of cytoskeletal components using stably transformed lines with dual-color reporters may provide a powerful tool for revealing coordinate behaviors and elucidating underlying mechanisms. VAEM observations on the dual-labeled epidermal cells demonstrated that cortical AFs and MTs were coaligned at numerous sites (Fig. 4a-b), which were mainly found between transversely or obliquely oriented AFs and MTs at the cell cortex. Straightening and bending events of cortical AFs, as reported previously [39], were also occasionally observed to occur within a couple of seconds at coalignment sites (Fig. 4b). Several studies have shown that perturbations of one cytoskeletal component by extracellular stimuli can change the organization of the other [18]. Here, we observed that the transverse MT organization gradually changed to a disordered configuration upon Pathogen-Associated Molecular Pattern (PAMP) (flg22 and chitin) exposure and resulted in a reduction of co-alignment between AFs and MTs (Fig. 4d-e). To quantify cytoskeletal remodeling in cotyledons, the cortical actin architecture was measured for density and skewness, which are metrics used for cytoskeletal component to estimate the percentage of occupancy and the extent of bundling, respectively [26, 40]. As shown in Fig. 4d-e and g, AF density in the cortical array was significantly increased after 5-min treatment with 1 μM flg22 or 10 μM chitin (*P* < 0.001), which was consistent with previous results for dark-grown hypocotyl cells [40]. However, MTs showed a significant decrease in density in the focus plane (P < 0.001), further suggesting that the MTs may also play a specific role in the perception of pathogenic microbes. Collectively, these data demonstrate that the cortical actin array in cotyledon epidermal cells responds within minutes to several diverse PAMPs, leading to significant increases in AF density. To examine the response specificity for the elicitor-triggered reorganization of cytoskeletal components as above, isoxaben, a cellulose synthesis inhibitor, was applied to the seedlings. As expected, following incubation the MTs were dramatically disordered and were changed into almost longitudinal fragments, further confirming the microtubule / microfibril paradigm; in contrast, actin filaments did not show significant changes in intensity and density (*P* > 0.05) (Fig. 4f-g). These results address the specificity of the coordinated behavior of AFs and MTs in response to PAMP elicitation, though the underlying mechanisms need further investigations.

### Comparative analysis of membrane compartmentalization by using LSCM and VAEM

**Fig. 5:**
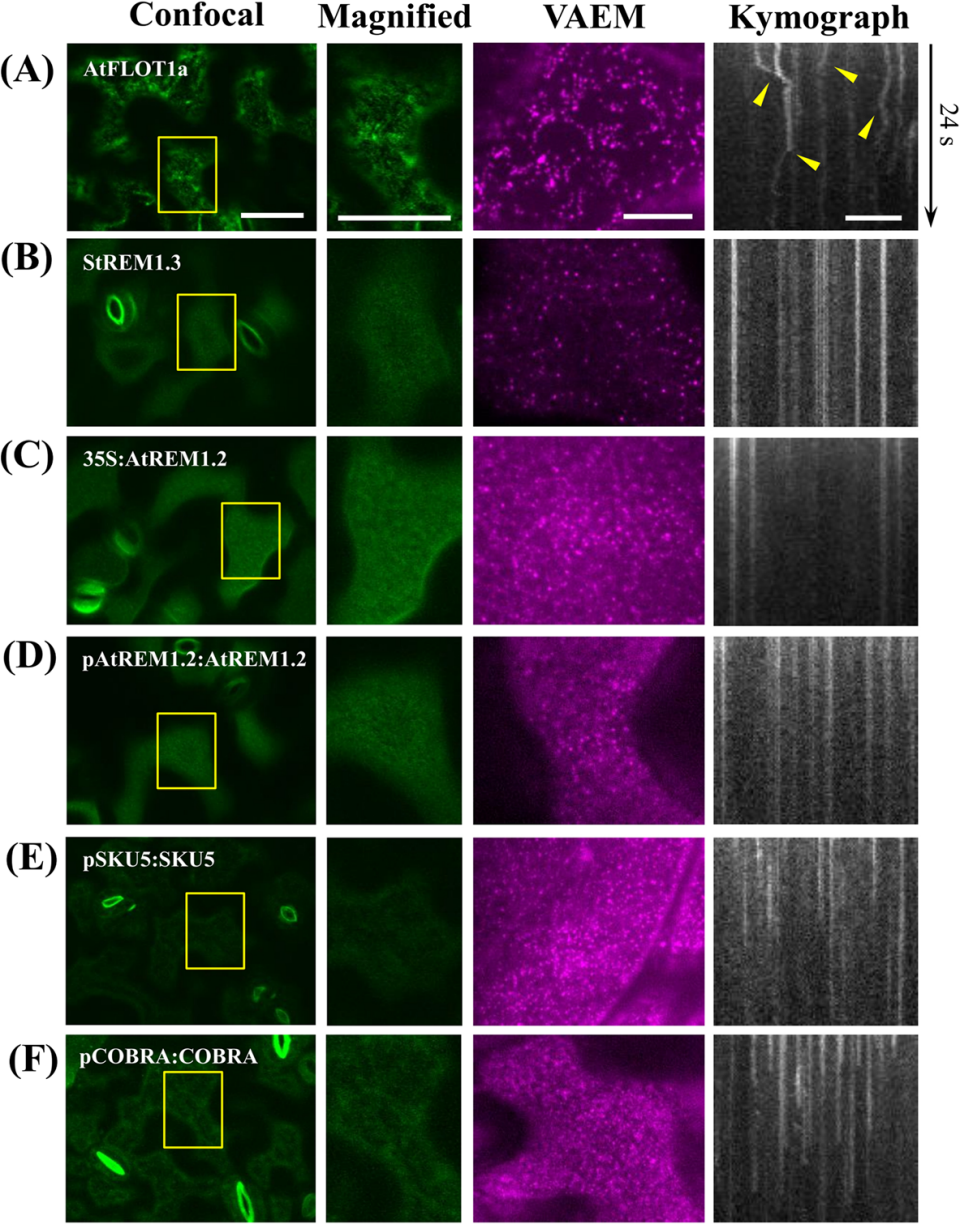
Comparative analysis on the PM subcompartmentation using LSCM and VAEM. To obtain a detailed view of the membrane domains, LSCM was firstly used to image the upper surface plane for cotyledon epidermal cells; VAEM and kymograph analysis were utilized to capture protein dynamics on the PM. **a** AtFlot1a displayed obviously uneven localization under LSCM and high lateral motility under VAEM, arrows indicate deviations in kymograph curves; (**b**-**d**) 35S:StREM, 35S:AtREM1.2, pREM1.2:AtREM1.2 showed diffused but uniformly distributed fluorescence labeling of the PM, while they formed puncta showing low lateral motility under VAEM; (**e**-**f**) pSKU5:SKU5-GFP and pCOBRA:COBRA-GFP demonstrated similar distribution patterns to those of REMs, but displayed relatively higher lateral motility. Bars = 50 μm (LSCM), 10 μm (VAEM); Interval between frames = 200 ms

As with yeast and animal cells, plant cells similarly have a sub-compartmentalized PM, in which membrane microdomains have been demonstrated to modulate endocytic events and signaling initiation [24, 41]. As shown in Fig. 5, transgenic *Arabidopsis* seedlings expressing several previously identified microdomain-located proteins (StREM1.3, AtREM1.2 and AtFLOT1a) were generated and examined under LSCM and VAEM, respectively. Two well-characterized glycosyl phosphatidylinositol (GPI)-anchored proteins, SKU5 and COBRA, were also selected, as it has been extensively reported in animal and in yeast cells that GPI proteins are predominantly located in membrane rafts [20, 42]. To obtain a detailed view of the membrane domains, LSCM was used to image the upper surface plane of cotyledon epidermal cells of 3- to 4-day-old plants. A diffuse but uniformly distributed fluorescence labeling of the PM was observed for the two previously identified remorins (REMs) and for the GPI-anchored proteins, however, microdomains showing significantly increased fluorescence intensity were not detected (Fig. 5b-f), while AtFLOT1a-GFP displayed an uneven localization on the cell surface (Fig. 5a).

By contrast, as VAEM allows observations at high signal-to-noise ratio (SNR) for cellular events occurring in the vicinity of the PM, both AtREM1.2 and StREM1.3 segregated into distinct microdomains showing relatively low motility in the time-lapse images as expected (Fig. 5b-d). Notably, GFP-AtREM1.2 as expressed under the control of the endogenous promoters formed static punctate structures as seen under VAEM (Fig. 5c-d), which were not significantly different from that expressed under the control of 35S promoter, indicating that *AtREM1.2* overexpression did not significantly change the expression pattern or the signal intensity in the membrane microdomains. To assess the temporal stability of these microdomains, time-lapse recording and kymograph analysis were performed on the time-series images. As expected, both SKU5 and COBRA displayed similar patterns in fluorescence distribution and punctate motility in comparison to REMs, since the results clearly showed that, for most of the punctate structures, REMs, SKU5, and COBRA all exhibited undetectable lateral stability (Fig. 5e-f). Interestingly, another previously identified microdomain-located protein, AtFLOT1a-GFP, displayed high motility and showed variability in punctate sizes, which may correspond to endocytic vesicles or different oligomerization statuses as previously reported for their homologs in mammalian cells (Fig. 5a) [14]. It did not localize to relatively stable microdomains in the PM, unlike StREM1.3 and AtREM1.2 as described above, further implying the heterogeneity of distinct microdomains and the biological complexity of membrane compartmentalization in vivo.

### Kinetic properties of membrane proteins as resolved by VAEM and single-particle tracking

**Fig. 6:**
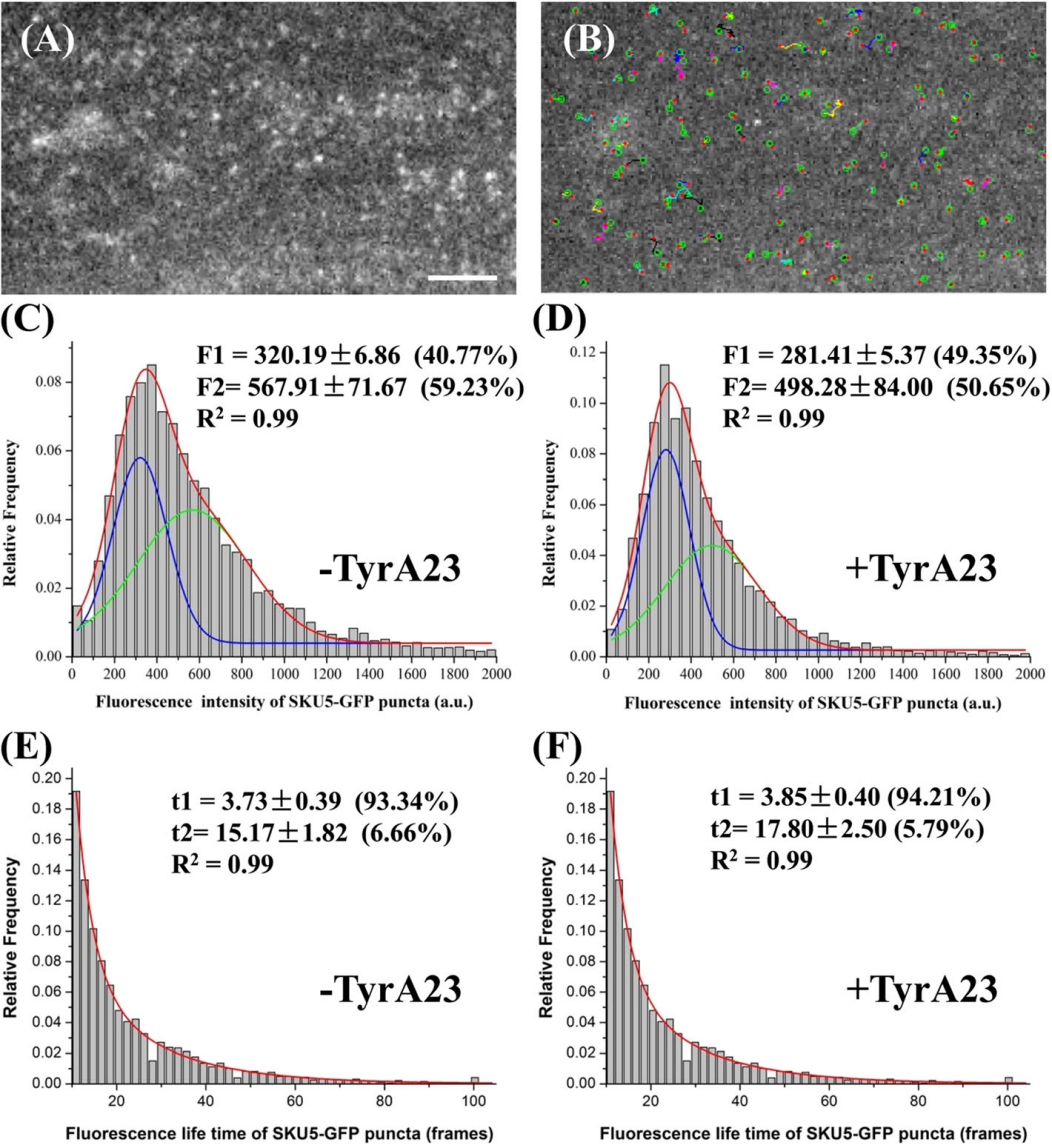
Single particle tracking and computational analysis on puncta of SKU5-GFP captured with VAEM. **a** A representative frame of time-lapse images for SKU5-GFP tagged puncta; **b** Trajectory of tracked puncta of SKU5-GFP-tagged puncta in panel (**a**); **c** The tracked SKU5-GFP tagged puncta were skewed to two populations in the histogram analysis based on fluorescence intensity after Gaussian fitting, implying different clustering pattern for the two populations; **d** TyrA23 treatment significantly altered the proportions of the two populations (*P* < 0.05); **e** The SKU5-GFP puncta were aligned to two populations in the histogram analysis based on life time after exponential fitting, implying different association states of the two populations; **f** The lifetime for two populations of SKU5-GFP puncta did not change significantly upon TyrA23 treatment (*P* > 0.05). Bars = 50 μm; *P* < 0.05, student t-test was carried out to examine the significance for the differences in the proportions between the two populations of SKU5-GFP puncta after tyrA23 treatment; Interval between frames = 200 ms

The PM provides sufficient spaces within which macromolecular interactions can take place efficiently, including the clustering of proteins in oligomeric complexes and the lateral diffusion of proteins. So far, among the currently identified GPI-anchored proteins in plant cells, SKU5-GFP has been experimentally demonstrated to be associated with the PM and localized to intracellular structures [20], which were found to be well co-localized with FM4–64-stained PM and vesicles (Additional file 2: Figure S6). The motility of membrane proteins is a critical determinant of their interaction capabilities and functions. Utilizing VAEM-based single-particle tracking, we were able to record and analyze the kinetic parameters representing the dynamics of SKU5-GFP puncta at single-particle level, including the fluorescence intensity, retention time, motion range, velocity, trajectory, and diffusion coefficient of individual puncta. As an example, the fluorescence intensity and retention time of individual puncta were analyzed with MATLAB software as examples (Fig. 6).

Notably, the SKU5-GFP puncta displayed different types of dynamic behavior, in which the fluorescence intensity and the retention time were two major parameters for characterizing the motility and oligomeric status of this GPI-anchored protein (Fig. 6a-b). The cumulative histograms for fluorescence intensity showed a skewed asymmetric distribution (*n* = 1813). Two-fifths (40.77%) of the particles had intensities ranging from 100 to 500 a.u., approximately the range for diffraction-limited monomeric GFP molecules in control cells (Additional file 2: Figure S7), suggesting that these particles were mostly composed of SKU5-GFP monomer. In contrast, the remaining puncta can be considered to exist as clusters composed of two or more SKU5 molecules since the cumulative histograms can be fitted into another Gaussian peak (59.23%) (Fig. 6c). In addition, the retention time (represented by the number of frames) for these particles, from their appearance to their disappearance on the membrane surface, ranged from 10 to 70 frames (2.3 s to 16.5 s), and the cumulative histograms of the lifetimes could be well fitted by a two-order exponential curve (τ1 = 3.73 ± 0.39, 93.34%; τ2 = 15.17 ± 1.82, 6.66%) (Fig. 6e). Moreover, the fit was not further improved with an increase to three exponential components, further implying that, in terms of their lifetime on the PM, SKU5-GFP molecules were largely present in the form of monomers or were organized into oligomers. To test whether clathrin-mediated endocytosis plays a role in membrane dynamics of SKU5-GFP, given that SKU5-GFP colocalized with FM4–64 in intracellular structures, pSKU5:SKU5-GFP seedlings were treated with 50 μM tyrphostin A23 (tyrA23), an inhibitor of clathrin-dependent endocytosis. It was found that, as a consequence, the proportions of the two subpopulations of pSKU5:SKU5-GFP puncta varied significantly (281.41 ± 5.37, 49.35%; 498.28 ± 84.00, 50.65%) (*n* = 1760, *P* < 0.05) compared to the control, while the retention time of SKU5-GFP puncta did not show significant changes (τ1 = 3.85 ± 0.40, 94.21%; τ2 = 17.80 ± 2.50, 5.79%) (*n* = 1760, *P* > 0.05) (Fig. 6d and f), further implying that clathrin-mediated internalization was involved in the membrane dynamics of both SKU5-GFP subpopulations. Importantly, TyrA23 significantly decreased the fluorescence intensity of both subpopulations and also the percentage of the subpopulation exhibiting higher fluorescence intensity (Fig. 6c-f). Further fluorescence correlation spectroscopy (FCS) analysis on fluorescence fluctuation also revealed a lower SKU5-GFP density (38.4 ± 4.1 molecules mm^− 2^; 14.9% decrease with respect to control cells at 44.6 ± 3.4 molecules mm^− 2^; *P* < 0.05, t-test) was detected after treatment with TyrA23 (Additional file 2: Figure S8).

**Fig. 7:**
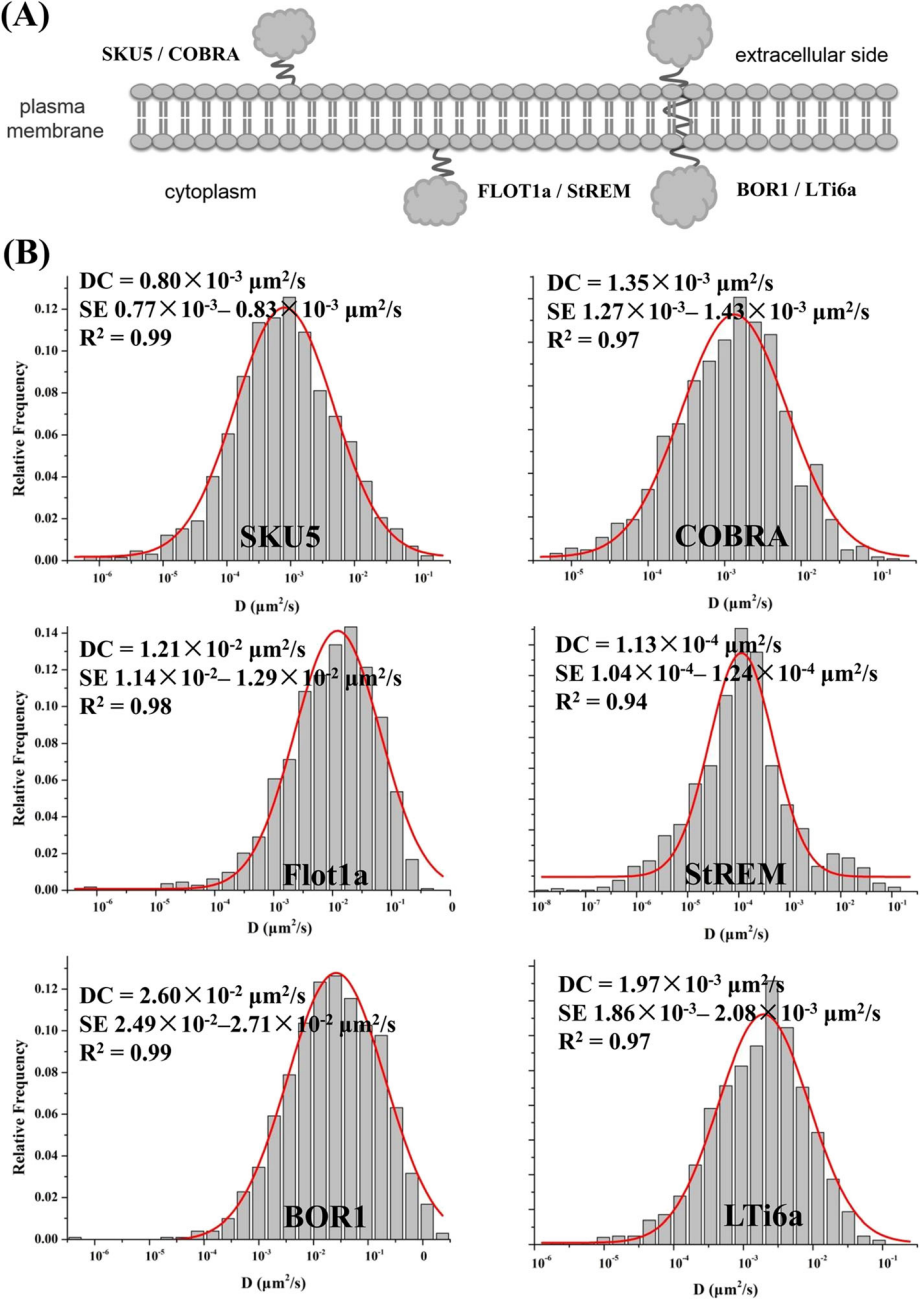
VAEM allows for analysis on dynamics of individual protein candidate at single particle level. Transgenic Arabidopsis line expressing pSKU5:SKU5-GFP, pCOBRA:COBRA-GFP, pBOR1:BOR1-GFP, 35S:LTi6a-GFP, 35S:GFP-Flot1a and 35S:GFP-StREM showing characteristic localization to punctate structures (**a**), which allows for further analysis on their dynamics on the PM, characterized by diffusion coefficient (DC) (**b**)

More than 25% of the proteome of higher plants is predicted to be membrane-associated proteins, including integral membrane proteins or peripheral membrane proteins according to the difference in their topology [43] (Fig. 7a). Considering this, we further selected SKU5 and COBRA, BOR1 and LTi6a, FLOT1a and StREM1.3, which are, respectively, well-characterized representatives of GPI-anchored proteins in the outer leaflet of the PM, transporters as representatives of integral membrane proteins, and proteins associated with the inner leaflet of the PM through post-translational modifications (such as S-acylation) respectively. As all of these proteins exhibited similar punctate structures varying in fluorescence intensity under VAEM, we then studied their dynamic properties in more detail by SPT and further analyzed the dynamic parameters using MATLAB software.

The diffusion coefficient (DC) is obtained from the trajectory of an individual particle, and the statistical distribution of single-trajectory diffusion coefficients may be useful as a measure of the heterogeneity of the membrane (Additional file 2: Figure S9) [28]. Both SKU5 and COBRA are well-characterized GPI-anchored proteins in plant cells, and they are expected to be preferentially located in membrane microdomains [41]. As a result, the diffusion coefficients of SKU5 and COBRA were distributed within one subpopulation of diffusion behavior (DC = 0.80 × 10^− 3^ μm^2^/s, *n* = 1321, SE 0.77 × 10^− 3^ – 0.83 × 10^− 3^ μm^2^/s, r^2^ = 0.99) (DC =1.35 × 10^− 3^ μm^2^/s, *n* = 1227, SE 1.27 × 10^− 3^– 1.43 × 10^− 3^ μm^2^/s, r^2^ = 0.97) (Fig. 7b). Similar to SKU5, the DC for LTi6a-GFP puncta was skewed to 1.97 × 10^− 3^ μm^2^/s (*n* = 1228, SE 1.86 × 10^− 3^– 2.08 × 10^− 3^ μm^2^/s, r^2^ = 0.97), which was comparable to the results obtained for the two GPI-anchored proteins; in contrast, the bona-fide endocytic cargo transmembrane protein BOR1-GFP was also distributed in a punctate manner as observed by VAEM [19]. The DC was 2.60 × 10^− 2^ μm^2^/s (*n* = 1013, SE 2.49 × 10^− 2^–2.71 × 10^− 2^ μm^2^/s, r^2^ = 0.99) (Fig. 7b), with a large dispersion of diffusion coefficients, most of which were between 2.49 × 10^− 2^ μm^2^/s and 2.71 × 10^− 2^ μm^2^/s, indicating that the motility of these proteins was heterogeneous, as also shown by kymograph analysis (Additional file 2: Figure S10). The resulting histograms were fitted by one or two Gaussian peaks, identifying as different subpopulations characterized by varying diffusion coefficients. In contrast, the inner-leaflet located AtFLOT1a displayed apparent high motility, and its DC was 1.21 × 10^− 2^ μm^2^/s (*n* = 1137, SE 1.14 × 10^− 2^– 1.29 × 10^− 2^ μm^2^/s, r^2^ = 0.98), further implying its potential involvement in endocytic events as previously reported. It was notable that the DC for StREM1.3-GFP was the lowest for all of the protein candidates examined, which together spanned a relatively broad range from 10^− 8^ μm^2^/s to 10^− 2^ μm^2^/s, with 1.13 × 10^− 4^ μm^2^/s (*n* = 1102, SE 1.04 × 10^− 4^– 1.24 × 10^− 4^ μm^2^/s, r^2^ = 0.94) as the median value; this further implied low motility for StREM1.3-GFP puncta and substantial scaffolding functions in the maintenance of membrane microdomains and in the initiation of signaling processes, as previously reported.

## Discussion

With recent advances in novel fluorescent proteins and imaging techniques, it is now feasible to visualize biological processes close to the PM at the subcellular level, or even at the single-particle level. TIRFM has been originally applied in plant research to in vitro studies on the AF and MT dynamics, as well as ER dynamics in protoplasts lacking cell wall [44]. Due to the exponential decay of the evanescent wave produced, the application of TIRFM to the study of plant and fungal cells is not straightforward, which is hampered by thick cell walls and poor adherence to glass surfaces. VAEM is applicable to different types of epidermal cells in plants. The first application of VAEM in plant cells can be retraced back to an attempt to follow secretory vesicular trafficking in lily pollen tubes, which originally described this technique as evanescent wave microscopy [8]. Konopka and Bednarek further defined the method as “variable-angle epifluorescence microscopy” (VAEM) to reduce background noise and to address some of the disadvantages of TIRF [9]. Mechanically, the only difference between performing microscopy in TIRF or VAEM mode is the change in orientation of the mirrors in the TIRF instrument, in which TIRF uses a single critical angle and VAEM ensures the use of a variety of subcritical angles. Therefore, no evanescence field but only a very thin band of illumination is generated in VAEM.

Basically, the close-up VAEM examination for cortical organelles may significantly facilitate the characterization of organelle identity, as shown in the dual-color labeling of intracellular structures in the present study. In terms of the controversy on the biological functions of ABP1, VAEM provides an alternative way to further explore the functional correlation of ABP1-tagged intracellular structures to endomembrane system. As ER acts as a gateway for the vast majority of proteins and lipids trafficking to various cellular compartments, the localization of ABP1 to ER implies potential roles of ABP1 at various contacting sites with other organelles. Von Wangenheim et al., reported that differential organization and motile behavior, and interactions of endosomes were related to particular root hair zones and developmental stages [45]. Interestingly, vesicular structures or endosomal compartments closely correlating with Golgi apparatuses may be actively involved in endocytic and exocytic activities in auxin signaling. Despite of the importance of local positioning, the precise connection between subcellular localization and organelle function is often not fully understood.

Vesicles are the smallest units between ER, Golgi and other endosomal compartments, and clathrin-dependent endocytosis has been extensively studies in plant cells [16, 32, 46]. Using VAEM, Konopka et al. compared dynamin-related protein 1 (DRP1) and clathrin dynamics in the cell cortex and further analyzed the functional redundancy of DRP1A and DRP1C during plant development. The results further advanced our understanding towards clathrin-mediated endocytosis and its regulation and confirming the robustness of VAEM in dissecting protein dynamics at single particle level in plant cells [16, 46]. Recently, AtFLOT1a was implied to be involved in a clathrin-independent endocytic event, which differs dramatically in size and dynamic properties from clathrin-coated vesicles [8]. Borrowing from the methodology adopted by Nakano lab in co-localizing clathrin and intracellular endosomes [32], we were able to categorize the numerous fluorescence-tagged CLC-GFP and FLOT1a puncta into different subpopulations in terms of the distance between individual puncta (Fig. 3). In the present study, the seemingly co-localized fluorescence signals of CLC-GFP and FLOT1a-mCherry under LSCM was confirmed to be not co-localized, but only partly associated indeed in the time-lapse observations with VAEM, as revealed by the results of overlapping coefficients and protein proximity index (Figs. 2 and 3). Noteworthy, overlapping coefficient has been well accepted as an index for evaluating the percentage of colocalization [32]. The sequential VAEM series can be directly imported for calculating overlapping coefficient (up to 10 sequential frames in the present study, depending on the computer memory), which is more precise and convincing for colocalization analysis in comparison to conventional LSCM.

Another advantage of VAEM is the imaging of fluorescently labeled structures closest to the coverslip [18, 44]. Tracking cytoskeletal components under VAEM may provide a powerful tool for resolving coordinate behaviors spatio-temporally. As narrated by Staiger’s lab in details, the AFs plays a central role not only in the physical organization of the cell, but also in the dynamic (re)localization of organelles, proteins, and macromolecules in response to pathogen infection [40, 47, 48]. Moreover, as tracks for the assembly and movement of defense-signaling components following pathogen perception, the AFs logically represents a virulence target for pathogens. However, a recent report shows that a type-III effector (T3E) protein from *Pseudomonas syringae*, HopZ1a, targets the MTs, thereby impairing the secretory pathway and suppressing cell wall-mediated defenses [49]. Similarly, AFs were stabilized through monoubiquitination of actin during infection by either pathogenic or mutualistic bacteria, but not in response to stress or viral infection [50]. This implies that the continuous rearrangements of the cytoskeleton within the same time scale may represent a surveillance mechanism to pathogens. In the present study, wild-type Col-0 plants showed significantly enhanced AF density following treatment with either flg22 or chitin, which may facilitate the endocytosis of pattern-recognition receptors (PRRs) and downstream signaling in plant cells [40]. The reorientation and fragmentation of MTs in response to PAMP stimuli may be attributed to the breakdown of interaction between cytoskeletal components, as PAMP decreased the frequency of co-alignment between AFs and MTs. In addition, super-resolution imaging methods, such as structural illumination microscopy (SIM), have also been applied in plant cell researches, e.g. microtubular dynamics in diverse cell types and correlation of RETICULONS to primary plasmodesmata and ER, which may further expand the potential of imaging methods in tackling key biological questions [51–54].

In terms of membrane subcompartmentalization, remorins (Rem) are above all the most bona-fide marker proteins for so-termed membrane raft or microdomain in the PM, as proved by recent advancements in identification of distinct functional microdomains in living cells [21, 24]. Here, we showed that StRem1.3, AtRem1.2 and the two GPI-anchored proteins were uniformly distributed on the PM in a diffused manner under LSCM, while further VAEM observations revealed relatively static punctate structures showing extremely low or only undetectable lateral motility on the PM, suggesting that microdomains for residing these proteins were temporally stable, as indicated by the vertical lines in the kymograph analysis. Similar results from fluorescence recovery after photobleaching (FRAP) experiments also revealed almost no photorecovery of DsRed2::AtRem 1.3 after photobleaching, indicating low mobility of these microdomain-resided proteins within the PM [21]. However, another previously reported microdomain-associated protein, FLOT1a [14], displayed obvious lateral motility, which may represent active endocytic events at the sites. These results may add additional values for investigating the heterogeneity of distinct microdomains and the complexity of their coexistence.

Proteins within membranes play crucial roles in signal perception and transduction, solute partitioning, and secretion [41, 55]. As the long-chain saturated lipid anchors found in many GPI-anchored proteins could facilitate their association with ordered-lipid microdomains or nanoclusters, we have been expecting that SKU5 and COBRA may display confined motility due to steric effects from the GPI-anchor, while integral proteins such as BOR1 may be confined to relatively limited areas. Notably, the diffusion coefficients of the proteins on the PM did not differ significantly, which were independent of the existence of membrane anchor. Therefore, GPI-anchor or other saccharide chains do not necessarily pose steric effects to influence motility parameters for protein dynamics in this case, while the properties of protein dynamics may directly correlate with their functions in specific biological context. Interestingly, Flot1a displayed relatively high lateral motility in comparison to other proteins examined, which was in accordance with previous reports on their functions in constitutive or ligand-induced endocytosis [14, 19]. It is also plausible that microdomains may act as structural units to physically separate proteins spatiotemporally and thus avoid of unexpected crosstalk in the absence of certain stimuli.

## Conclusion

In summary, VAEM offers a powerful method for probing protein dynamics and other intracellular events in the proximity of the PM and the cell cortex. It may further advance our insights into membrane trafficking, cytoskeletal organization and membrane subcompartmentalization in specific contexts with improved spatial and temporal precision. With the widespread utility and increasing accessibility of VAEM, SPT and computational methodology, the future promises excitement for those interested in revealing how protein dynamics, interactions, and functions define cell-specific profiles in response to developmental cues or environmental stimuli.

## Additional files

Additional file 1: Methodology for determining incident angle and penetration depth. (PDF 569  kb)

Additional file 2: **Figure S1.** Organellar dynamics are resolved in high spatio-temporal manner under VAEM. **Figure S2.** VAEM is applicable to epidermal cells of different tissues from seedlings. **Figure S3.** CLC-GFP tagged punctate structures correspond to clathrin-coated vesicles and Golgi apparatuses, depending on the angle of incident light. **Figure S4.** Actin turnover resolved using VAEM. **Figure S5.** Microtubular organization as revealed by VAEM. **Figure S6.** SKU5-GFP localizes to plasma membrane and intracellular structures. **Figure S7.** Distribution of the fluorescence intensity of the diffraction-limited single pCLC2- myristoyl-mGFP^A206K^ spots. **Figure S8.** Fluorescence correlation spectroscopy (FCS) to examine the fluorescence fluctuation in response to TyrA23 treatment. **Figure S9.** Analysis on MSD for different trajectories and categorization into various diffusion regimes. **Figure S10.** Representative kymographs for BOR1 and Lti6a. (PDF 1148 kb)

Additional file 3: Dynamics of Golgi apparatuses in the proximity of plasma membrane. (AVI 23044 kb)

Additional file 4: Dynamics of mitochondria in the proximity of plasma membrane. (AVI 23044 kb)

Additional file 5: Dynamics of endoplasmic reticulum (ER) tubules in the proximity of plasma membrane. (AVI 23044 kb)

Additional file 6: Dual-color VAEM indicates clathrin light chain (CLC)–GFP and mCherry-Flot1a showing characteristic localization to separated punctate structures. (AVI 127 kb)

Additional file 7: Rapid turnover of actin filaments in transgenic fABD2-GFP Arabidopsis seedlings in the proximity of plasma membrane. (AVI 1151 kb)

Additional file 8: Time-lapse recording for microtubular organization as revealed by VAEM. (AVI 1072 kb)

## Abbreviations

a.u.: Arbitrary unit
ABP1: Auxin binding protein 1
AFs: Actin filaments
At: *Arabidopsis thaliana*
BFA: Brefeldin A
BOR1: Boron transporter 1
CLC: Clathrin light chain
DC: Diffusion coefficient
DRP1C: dynamin related protein 1C
EMCCD: Electron multiplying charge coupled device
ER: Endoplasmic reticulum
fABD2: Filamentous-actin binding domain of fimbrin2
FCS: Fluorescence correlation spectroscopy
FLOT1a: FLOTTILIN1a
FRAP: Fluorescence recovery after photobleaching
GFP: Green fluorescent protein
GPI: Glycosyl phosphatidylinositol
HiLo microscopy: Highly inclined and laminated optical sheet microscopy
LSCM: Laser scanning confocal microscopy
LTi6a: Low temperature induced 6a
mGFP: Monomeric green fluorescent protein
mRFP: Monomeric red fluorescent protein
MSD: Mean square displacement
MTs: Microtubules
PM: Plasma membrane
PRRs: Pattern-recognition receptors
REMs: Remorins
SIM: Structural illumination microscopy
St: *Solanum tuberosum*
T3E: Type-III effector
TGN: Trans-Golgi network
TIRF: Total internal reflection fluorescence
TUA5: Tubulin alpha 5
VAEM: Variable-angle epifluorescence microscopy
WCIF: Wright cell imaging facility
