# Supplementary material for: Variable-angle epifluorescence microscopy characterizes protein dynamics in the vicinity of plasma membrane in plant cells
Source: BMC Plant Biol. 2018 Mar 14;18:43. doi: 10.1186/s12870-018-1246-0 (PMC5853057; doi:10.1186/s12870-018-1246-0)
Supplement: Supplementary file 1 — Methodology for determining incident angle and penetration depth. (PDF 569 kb) [file 12870_2018_1246_MOESM1_ESM.pdf]

Additional file 1

Methodology for determining incident angle and penetration depth

We previously presented theoretical and experimental evidence for the application of TIRF and VAEM in plant cells in our work published in *Plant Methods* (Wan *et al*., 2011). Under TIRF conditions, all of the light is reflected back into the medium with the higher n value (refractive index) (Formula 1) (Fish, 2009). When TIR occurs, a nearfield light wave forms at the boundary; this "evanescent wave" (EW) can penetrate the surface of the medium to a depth (d) approximately equal to 1/3 of the wavelength of the incident light (Formula 2) (Fish, 2009).

Formula for calculating critical angle of incidence, in which θ_i stands for the incident angle and θ_c stands for the critical angle of incidence.

$$\theta_c = \sin^{-1} (n_1/n_2) \qquad (1)$$

Formula for calculating the depth of an EW field:

$$d = \lambda_0/4\pi(n_2^2\sin^2\theta - n_1^2)^{-1/2} \qquad (2)$$

In terms of VAEM, it allows light to penetrate the cell wall using a sub-critical angle, in which θ_i is slightly smaller than θ_c (Konopka and Bednarek, 2008). In our VAEM system, we coupled the TIRF module to a device with which the θ_i is continuously adjustable. The microscopic analysis on plant cells must accommodate five different peripheral layers between the incident light and the cytosol (n=1.38); these layers are the immersion oil (n=1.52), coverslip (n=1.52), aqueous medium (n=1.33), cell wall (n=1.42 to 1.48), and plasma membrane (d=7 nm) (Fish, 2009; Woolley, 1975).

According to Formula 1, θ_c at the g/am interface is 61.0°. The angle of refraction (δ) inside the cytosol for a sub-critical angle of incidence ($\theta_i < \theta_c$) can be calculated using Snell's law (Formulas 3 and 4). With the n values given above for cytosol and glass and θ_i set at 60.0° (slightly less than θ_c), then δ is 72.5° inside the cytosol. The depth of the illumination field from the refractive laser light can then be calculated by a trigonometric formula (Formula 5).

Snell's law describes the relationship between θ_i and δ as:

$$\sin\theta_i / \sin\delta = n_2/n_1 \qquad (3)$$

When incident light penetrates × different optical layers, δ in the last medium (δ_x) can be calculated as:

$$\sin\theta_i / \sin\delta_x = n_x/n_1 \qquad (4)$$

Variable depth of illumination (d) within an observation field (width = a) can be calculated as:

$$d = a / \tan\delta x \qquad (5)$$

In our VAEM experiments, the visual field of the EM-CCD camera is 512×512 pixels, corresponded to 81.9×81.9 μm (a=81.9 μm); according to formula 5, d varies from 0 to 25.9 μm in this field. Given that the width of a single cell is 20 μm, d may vary from 0 to 6.3 μm.

Regarding the n values of the different peripheral layers in plant cell microscopy, TIR may occur at two different interfaces, the cell wall/cytoplasm (cw/c) interface and the glass/cell wall (g/cw) interface. Given that the average n of cell walls is 1.45 and that cell walls of epidermal cells are smooth and in uniform thickness (roughly 250 nm), θ_c at the cw/c interface ($\theta_{c(cw/c)}$) is 72.1°. According to Snell's law (Formula 3), θ_i from the objective lens (θ_{c1} in panels C and E) should be greater than 65.2° to create TIR at the cw/c interface. When θ_i is further increased by the variable angle device and reaches θ_c at the g/cw interface ($\theta_{c(g/cw)}$ = 72.5°), the light beam is completely reflected back (θ_{c2} in panel D). Therefore, there are two critical requirements to obtain EW field illumination inside the cytosol in an intact plant cell: an incident angle between θ_{c1} and θ_{c2} (65.2° < θ_i < 72.5°) and a sufficiently thin aqueous film (d < 100 nm). When the EW field occurs inside the cytosol of an intact plant cell, the depth of illumination is constant for a single observation (panel E).

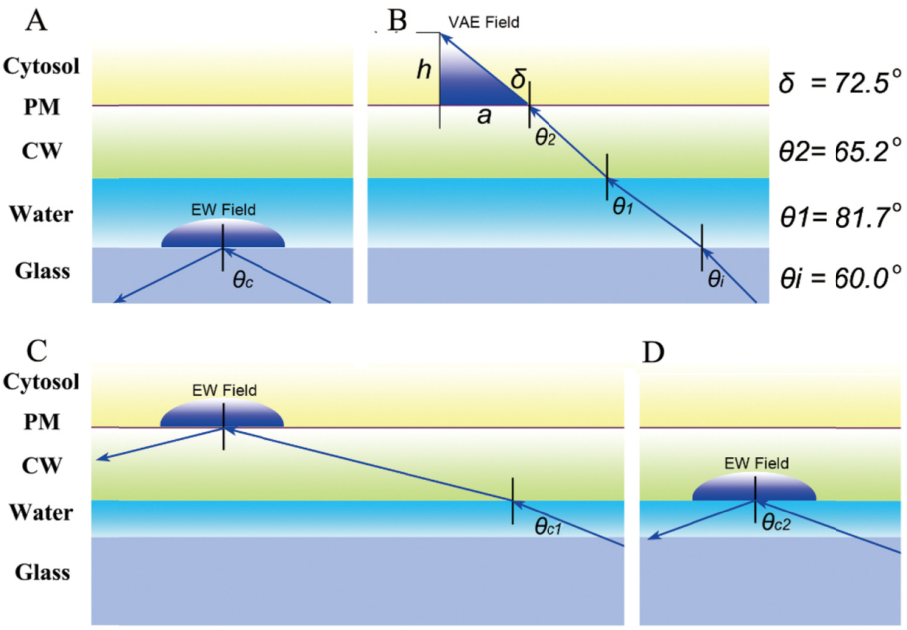

E

	Refractive index	Thickness (nm)	Critical angle	Depth of EW
Glass	1.52	140 µm		-
Water	1.33	>100nm	θ_c : 61.0°	105 nm
Water		<100nm	-	-
Cell Wall	1.42-1.48	250nm	θ_{c1} : 72.5°	-
Cytosol	1.38	-	θ_{c2} : 65.2°	159nm

Optical analysis of light paths in VAEM and TIRFM observations in plant cells.

(Wan *et al*., 2011)

Given the theoretical value for incident angles in VAEM observations, we further measured the incident angles using transgenic Arabidopsis CLC-GFP, HDEL-GFP and SYP22-GFP seedlings (clathrin, ER marker and tonoplast marker). In the present study, a micrometer allowing continuous lateral adjustment of the spatial filter assembly was equipped on the TIRF system, and hence the position of the beam at the back aperture of the objective can be modulated to switch between TIRF and epifluorescence modes. We changed the θ_i to keep $\theta_{c1} < \theta_i < \theta_{c2}$ and gently pressed the seedling against the surface of the glass slide to obtain better adherence. The results showed that the incident angles were between 63.03° and 66.64° for HDEL-GFP and SYP22-GFP seedlings, which corresponded to two critical statuses for VAEM

observations on intracellular structures just beneath the PM. Judging from the curve incorporated in the cell TIRF software, the penetration depths corresponding to the two angles were 120 nm (66.64°) – 250-300 nm (63.03°) respectively, depending on the practical conditions for the adherence of seedlings. For CLC-GFP seedlings, the incident angles were between 65.69° and 67.73°, the penetration depths corresponding to the two angles were 100 nm (67.73°) and 130 nm (65.69°).

References

Fish K. (2009) Total internal reflection fluorescence (TIRF) microscopy. *Current Protocol in Cytometry*, **12**, Unit 12.18.

Ash W.M. III, Krzewina L., Kim M.K. (2009) Quantitative imaging of cellular adhesion by total internal reflection holographic microscopy. *Applied Optics*, **48**, 144–152.

Konopka, C.A. and Bednarek, S.Y. (2008) Variable-angle epifluorescence microscopy: a new way to look at protein dynamics in the plant cell cortex. *Plant Journal*, **53**, 186–196.

Wan Y.L., Ash W.M. III, Fan L.S., Hao H.Q., Kim M.K., Lin J.X. (2011) Variable-angle total internal reflection fluorescence microscopy of intact cells of *Arabidopsis thaliana*. *Plant Methods*. **7**, 27.

Woolley J.T. (1975) Refractive index of soybean leaf cell walls. *Plant Physiology*, 55, 172–174.
