# Supplementary material for: Variable-angle epifluorescence microscopy characterizes protein dynamics in the vicinity of plasma membrane in plant cells
Source: BMC Plant Biol. 2018 Mar 14;18:43. doi: 10.1186/s12870-018-1246-0 (PMC5853057; doi:10.1186/s12870-018-1246-0)
Supplement: Supplementary file 2 — Figure S1. Organellar dynamics are resolved in high spatio-temporal manner under VAEM. Figure S2. VAEM is applicable to epidermal cells of different tissues from seedlings. Figure S3. CLC-GFP tagged punctate structures correspond to clathrin-coated vesicles and Golgi apparatuses, depending on the angle of incident light. Figure S4. Actin turnover resolved using VAEM. Figure S5. Microtubular organization as revealed by VAEM. Figure S6. SKU5-GFP localizes to plasma membrane and intracellular structures. Figure S7. Distribution of the fluorescence intensity of the diffraction-limited single pCLC2- myristoyl-mGFPA206K spots. Figure S8. Fluorescence correlation spectroscopy (FCS) to examine the fluorescence fluctuation in response to TyrA23 treatment. Figure S9. Analysis on MSD for different trajectories and categorization into various diffusion regimes. Figure S10. Representative kymographs for BOR1 and Lti6a. (PDF 1148 kb) [file 12870_2018_1246_MOESM2_ESM.pdf]

Variable-Angle Epifluorescence Microscopy Characterizes Protein Dynamics in the Vicinity of Plasma Membrane in Plant Cells

Tong Chen [a,1], Dongchao Ji [a,b,1], Shiping Tian [a,b,c]

[a] Key Laboratory of Plant Resources, Institute of Botany, Chinese Academy of Sciences, Beijing 100093, China

[b] University of Chinese Academy of Sciences, Beijing 100049, China

[c] Key Laboratory of Post-Harvest Handling of Fruits, Ministry of Agriculture, China

[1]These authors contribute equally to this work

Figure S1

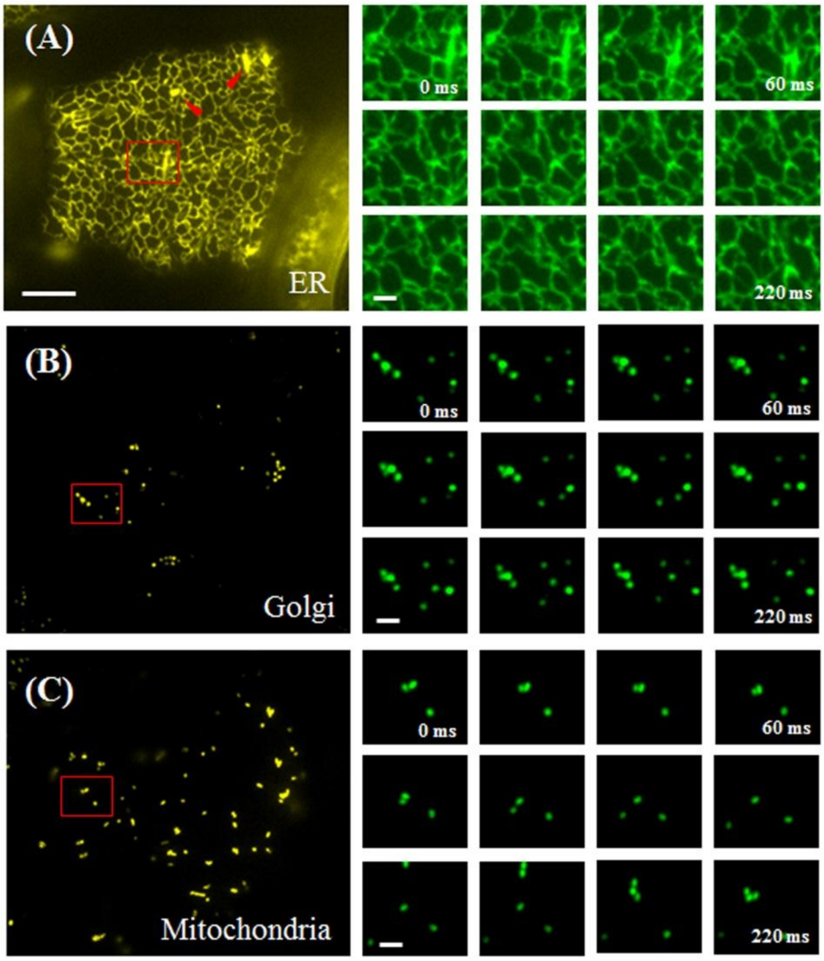

Figure S1 Organellar dynamics are resolved in high spatio-temporal manner under VAEM. Areas in the brackets are shown in time series in the right.

(A) Transgenic *Arabidopsis* line expressing HDEL-GFP (35S:HDEL-GFP / Col-0) showing endoplasmic reticulum (ER) in the proximity of the PM in high spatio-temporal dynamics, arrows indicates ER bodies;

(B) Transgenic *Arabidopsis* line expressing NAG-GFP (35S:NAG-GFP / Col-0) showing accumulated Golgi apparatuses displaying relatively low motility;

(C) Transgenic *Arabidopsis* line expressing mCherry-ssβATPase (35S: mCherry-ssβATPase / Col-0) showing frequent tethering and detaching activities of mitochondria.

The time series are shown for every 10 images; Bars = 10 μm (panels in the left), 2.5 μm (time series in the right); Interval between frames = 20 ms.

Figure S2

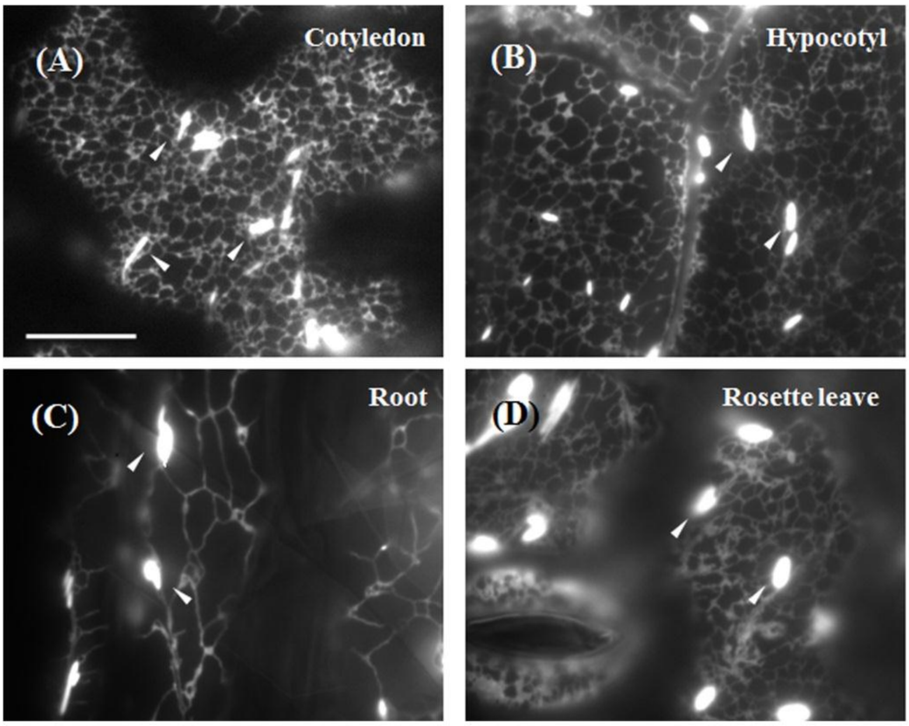

Figure S2 VAEM is applicable to epidermal cells of different tissues from seedlings

(A) Transgenic *Arabidopsis* line expressing HDEL-GFP showing characteristic localization to ER laminar and ER bodies in cotyledon, arrows indicates ER bodies;

(B) Transgenic *Arabidopsis* line expressing HDEL-GFP showing similar pattern in hypocotyl to that in cotyledon;

(C) Transgenic *Arabidopsis* line expressing HDEL-GFP showing relatively stable ER network in root.

(D) Freshly detached slice from rosette leave showing characteristic localization to ER laminar and ER bodies.

Bars = 10 μm; Arrows indicate ER bodies; Interval between frames = 20 ms.

Figure S3

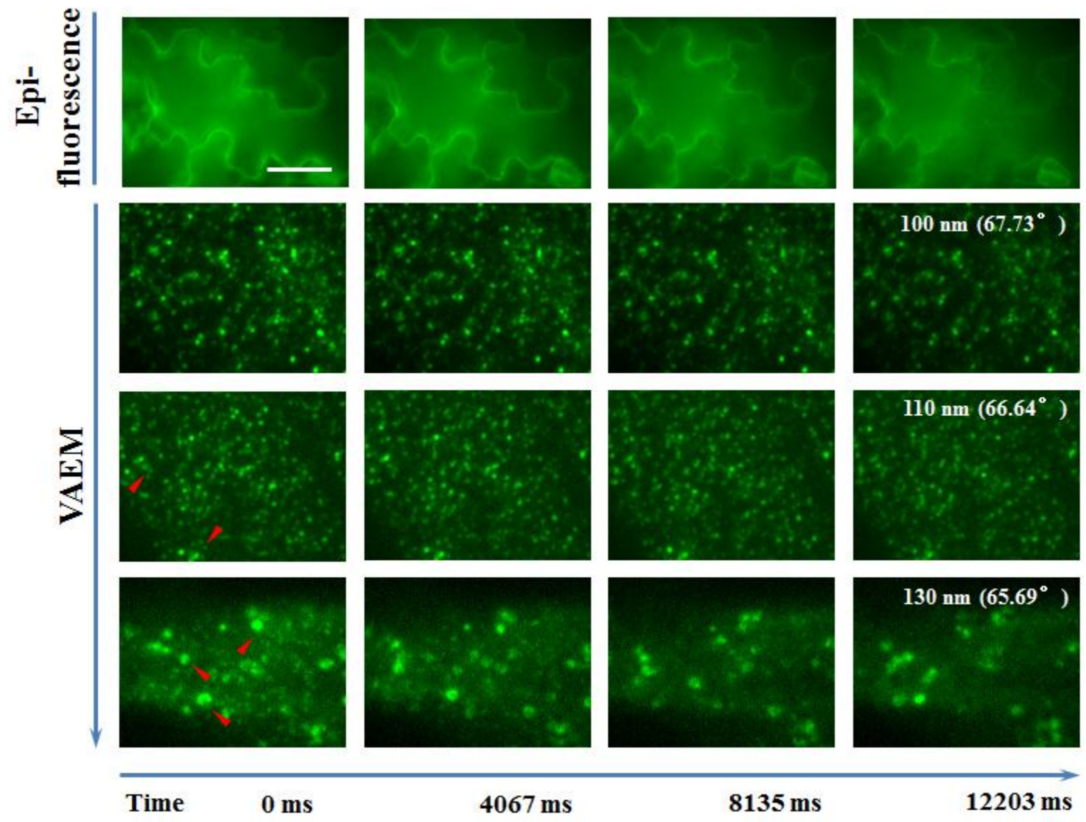

Figure S3 CLC-GFP tagged punctate structures correspond to clathrin-coated vesicles and Golgi apparatuses, depending on the angle of incident light.

Transgenic Arabidopsis line expressing clathrin light chain (CLC) -GFP showing characteristic localization to highly dynamic punctate structures, which show low motility in the very proximity of PM. Corresponding penetration depths and incident angles were shown in the right up corners for the VAEM images.

Bars = 7 μm; longitudinal axis indicates the depth of illumination light into cells, depending on the angle of incident light, while latitudinal axis indicates the time points at which the images are captured (the images are shown for every 20 frames). Interval between frames = 100 ms.

Figure S4

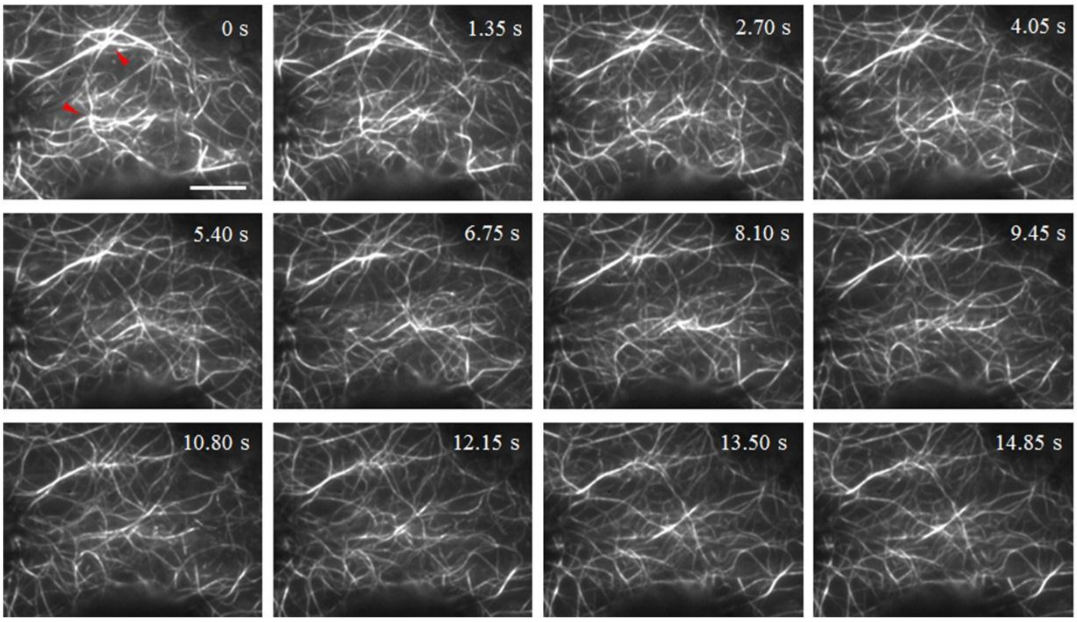

Figure S4 Actin turnover resolved using VAEM.

Transgenic Arabidopsis line expressing fABD2-GFP showing characteristic localization to AFs, which is characterized by rapid turnover;

Bars = 10 μm; arrows indicate sites of AFs undergoing bundling or depolymerizing activities; Interval between frames = 200 ms.

Figure S5

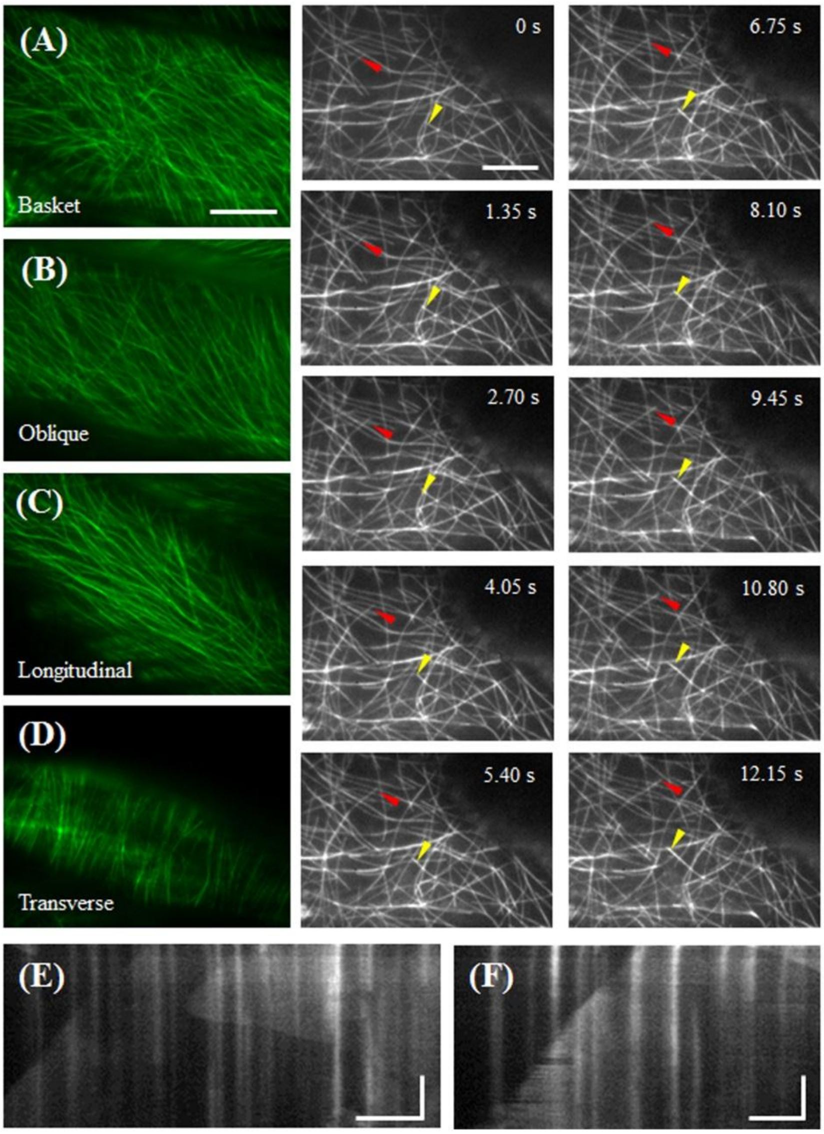

Figure S5 Microtubular organization as revealed by VAEM

Transgenic Arabidopsis line expressing α-tubulin 5 isoform (TUA5)-mCherry showing all the four previously reported organization patterns in hypocotyl: basket, oblique, longitudinal and transverse; the microtubules were more stable in comparison to actin filaments.

Bars = 10 μm; Interval between frames = 200 ms.

Figure S6

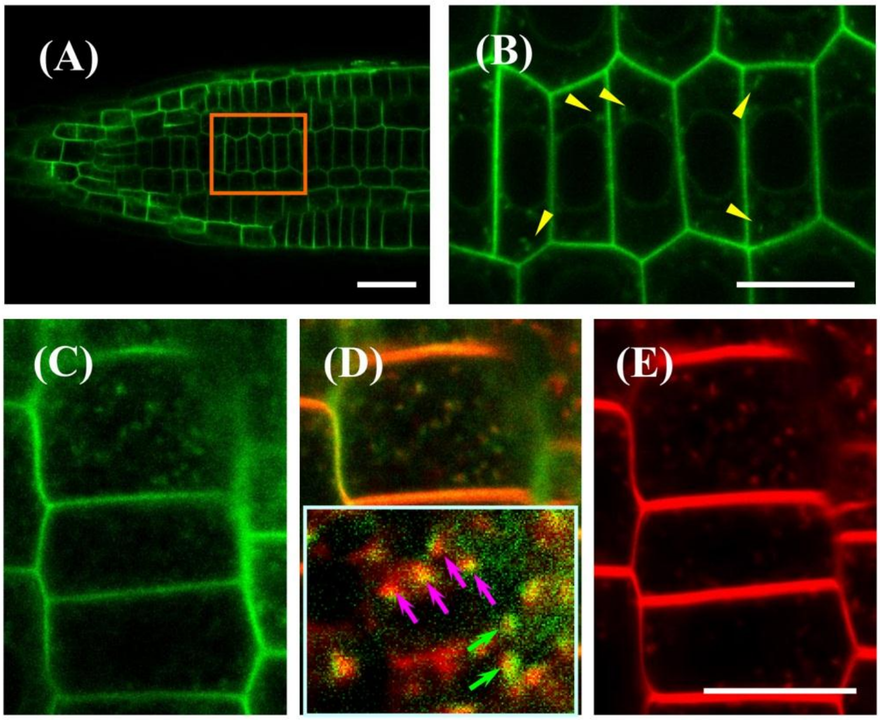

Figure S6 SKU5-GFP localizes to plasma membrane and intracellular structures

(A) Transgenic Arabidopsis line expressing pSKU5-SKU5-GFP showing characteristic localization to plasma membrane in root;

(B) Close-up examinations indicate pSKU5-SKU5-GFP also target to intracellular structures;

(C-E) Punctate structures of pSKU5-SKU5-GFP co-localizes to internalized FM4-64 signals. The inlet showed colocalized SKU5 puncta and FM4-64 puncta.

Bars = 25 µm (A), 10 µm (B-E); Arrows indicate punctate structures of pSKU5-SKU5-GFP in cytoplasm.

Figure S7

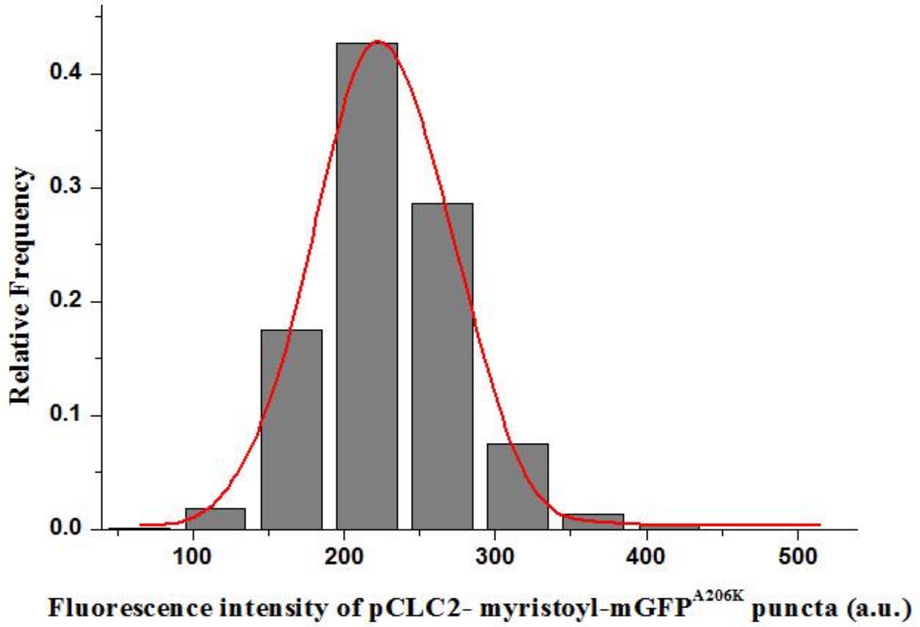

Figure S7 Distribution of the fluorescence intensity of the diffraction-limited single pCLC2- myristoyl-mGFP^{A206K} spots (n = 1500)

The single particle tracking and image analysis were carried out in five cells from each of five representative Arabidopsis roots

Figure S8

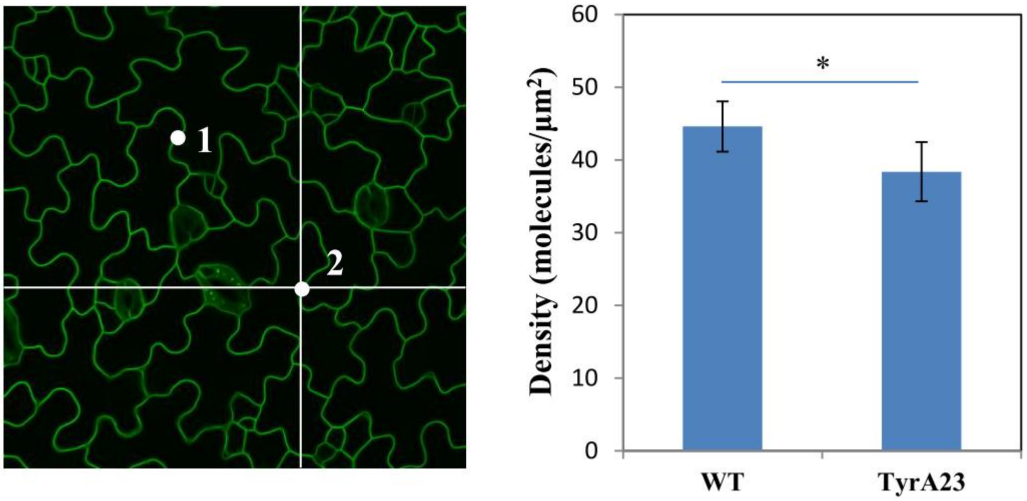

Fig. S8 Fluorescence correlation spectroscopy (FCS) to examine the fluorescence fluctuation in response to TyrA23 treatment.

A lower SKU5-GFP density (40.3 ± 4.1 molecules mm^{-2}; 9.7% decrease with respect to control cells; * $P < 0.05$, t-test) was detected after treatment with TyrA23, indicating that less SKU5-GFP molecules dwell on the membrane after the inhibition of clathrin-dependent endocytosis.

Fig. S9

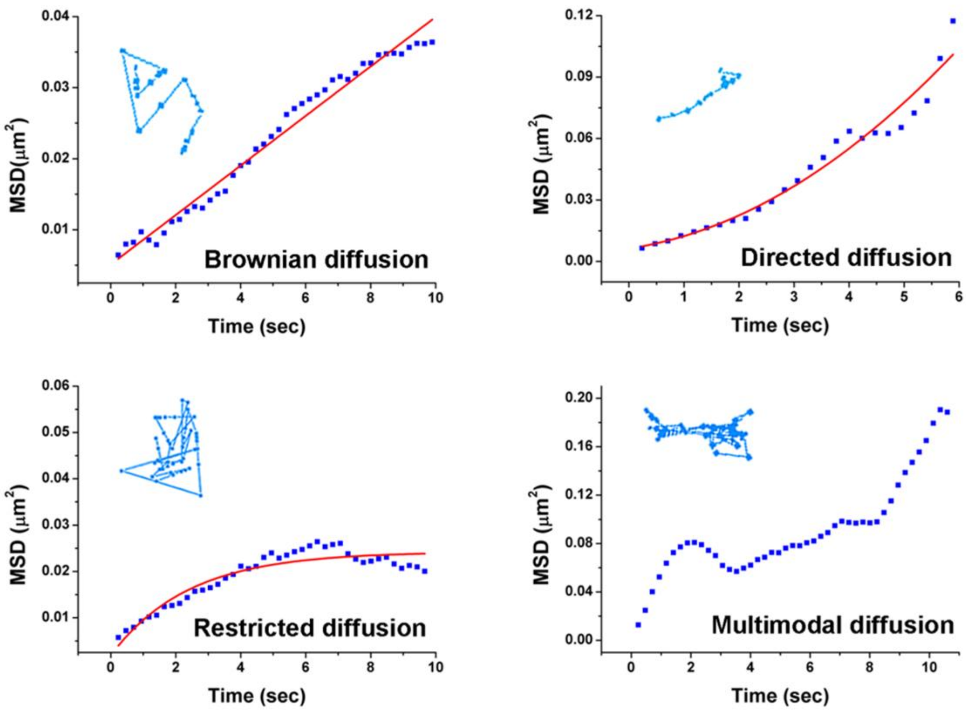

Fig. S9 Analysis on MSD for different trajectories and categorization into various diffusion regimes.

The MSD-*t* curves (blue lines in the left above corner) were fitted with pure Brownian, directed, restricted and multimodal diffusion. A representative sample of different diffusion modes of SKU5-GFP puncta was shown with trajectories.

Fig. S10

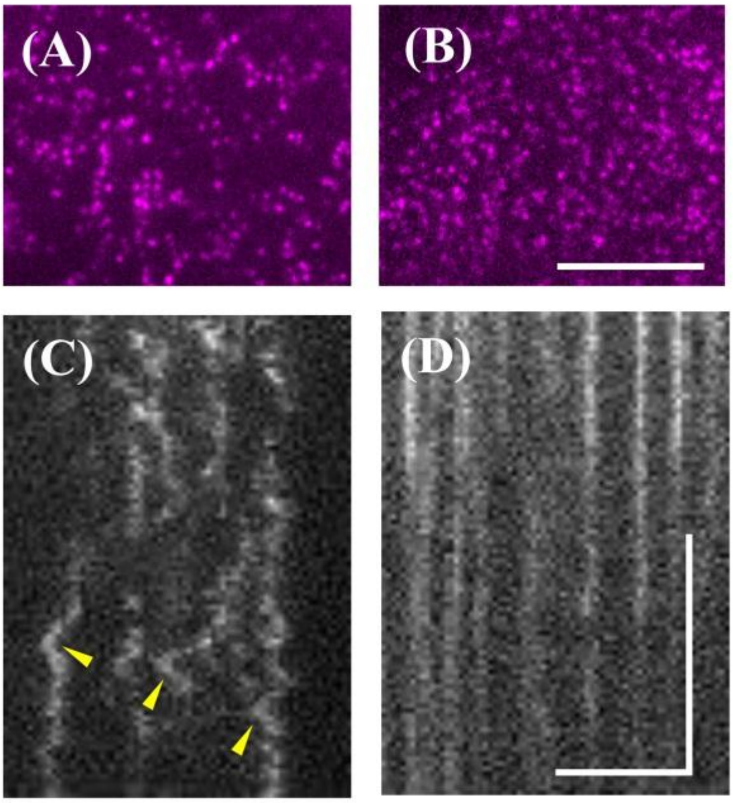

Fig. S10 Representative kymographs for BOR1 and Lti6a

(A) 35S:BOR1-GFP formed puncta showing lateral motility under VAEM;

(B) 35S:LTi6a-GFP formed puncta showing low lateral motility under VAEM;

(C) Kymograph analysis for 35S:BOR1-GFP puncta indicated higher motility;

(D) Kymograph analysis for 35S:LTi6a-GFP;

Interval between frames = 100 ms. In all kymographs (C-D), time evolution is from top to bottom. The time bar in the kymographs (d) represent 12 s. Bars = 10 μm (A, B), 3 μm (C, D).
